# Impact of multi-component school food environment interventions on adiposity and food consumption in children and adolescents: systematic review and meta-analysis

**DOI:** 10.1590/0102-311XEN152824

**Published:** 2025-12-01

**Authors:** Luisa Arantes Vilela, Camila Kümmel Duarte, Luana Lara Rocha, Brenda da Cunha Carvalho, Ariene Silva do Carmo, Lúcia Helena Almeida Gratão, Thales Philipe Rodrigues da Silva, Milene Cristine Pessoa, Larissa Loures Mendes

**Affiliations:** 1 Universidade Federal de Minas Gerais, Belo Horizonte, Brasil.; 2 Universidade Federal de Ouro Preto, Ouro Preto, Brasil.; 3 Universidade Federal de São Paulo, São Paulo, Brasil.

**Keywords:** School Feeding, School Health Services, Nutrition Policy, Nutrition Programs, Noncommunicable Diseases, Alimentação Escolar, Serviços de Saúde Escolar, Política Nutricional, Programas de Nutrição, Doenças não Transmissíveis, Alimentación Escolar, Servicios de Salud Escolar, Política Nutricional, Programas de Nutrición, Enfermedades no Transmisibles

## Abstract

Childhood overweight and obesity are growing public health concerns, leading to metabolic consequences such as increased body mass index, larger waist circumference, and excess body fat. Multi-component school interventions that address both the obesogenic environment and individual behaviors have been recommended, but their effectiveness remains uncertain. This review and meta-analysis, conducted following PRISMA guidelines, examined the impact of multi-component interventions - including modifications to the school food environment - on adiposity and food consumption among children and adolescents. A search on MEDLINE, SciELO, CENTRAL, Clinical Trials, Scopus, Embase, and Web of Science identified 51 eligible studies. The meta-analysis showed a small but significant reduction in waist circumference (MD: -0.70cm; 95%CI: -1.22, -0.19; I^2^ = 40%). Interventions were also linked to lower intake of unhealthy foods, total energy, total fat, saturated fat, and increased vegetable consumption. However, no consistent effects were observed for body mass index or body fat percentage. Study quality varied, and intervention designs and implementation strategies were heterogeneous; thus, results should be interpreted cautiously. These findings suggest that while school food environment interventions can improve some dietary behaviors and adiposity indicators, their effectiveness in preventing obesity remains inconclusive. Strengthening policies and ensuring long-term, structured interventions are crucial for meaningful and sustained health improvements in school settings.

## Abstract (pt)

Sobrepeso e obesidade infantis representam desafios crescentes para a Saúde Pública, levando a consequências metabólicas, como aumento do índice de massa corporal, maior circunferência da cintura e excesso de gordura corporal. Recomendam-se intervenções escolares multicomponentes que integram fatores relacionados ao ambiente obesogênico e aos comportamentos individuais, mas sua eficácia permanece incerta. Esta revisão e metanálise, conduzida seguindo as diretrizes PRISMA, focou no impacto de intervenções multicomponentes - que incluíram mudanças no ambiente alimentar escolar - na adiposidade e no consumo alimentar de crianças e adolescentes. A busca nas bases de dados MEDLINE, SciELO, CENTRAL, Clinical Trials, Scopus, Embase e Web of Science identificou 51 estudos elegíveis. A metanálise mostrou uma redução pequena, mas significativa, na circunferência da cintura (DM: -0,70cm; IC95%: -1,22, -0,19; I^2^ = 40%). As intervenções também se associaram à menor ingestão de alimentos não saudáveis, energia total, gordura total e gordura saturada, bem como um aumento do consumo de hortaliças. No entanto, não foram observados efeitos consistentes no índice de massa corporal ou no percentual de gordura corporal. A qualidade dos estudos variou, com heterogeneidade no desenho e na implementação das intervenções. Portanto, os resultados devem ser interpretados com cautela. Os achados deste estudo sugerem que, embora intervenções no ambiente alimentar escolar possam levar à melhora de alguns comportamentos alimentares e medidas de adiposidade, seu impacto na prevenção da obesidade permanece inconclusivo. O fortalecimento de políticas e a garantia de intervenções estruturadas de longo prazo são cruciais para alcançar melhoras significativas e sustentadas na saúde em ambientes escolares.

## Abstract (es)

El sobrepeso y la obesidad infantil representan un creciente desafío para la Salud Pública, con consecuencias metabólicas como el aumento del índice de masa corporal, una mayor circunferencia de cintura y un exceso de grasa corporal. Se han recomendado intervenciones escolares multicomponentes que integren factores relacionados con el entorno obesogénico y los comportamientos individuales, pero su efectividad sigue siendo incierta. Esta revisión y metaanálisis, realizado siguiendo las directrices PRISMA, se centró en el impacto de intervenciones multicomponentes que incluyeron cambios en el entorno alimentario escolar, sobre la adiposidad y el consumo de alimentos en niños y adolescentes. Una búsqueda en MEDLINE, SciELO, CENTRAL, Clinical Trials, Scopus, Embase y Web of Science identificó 51 estudios elegibles. El metaanálisis mostró una reducción pequeña pero significativa en la circunferencia de cintura (DM: -0,70cm; IC95%: -1,22, -0,19; I^2^ = 40%). Las intervenciones también se asociaron con un menor consumo de alimentos no saludables, energía total, grasa total, grasa saturada y un aumento en el consumo de verduras. Sin embargo, no se observaron efectos consistentes en el índice de masa corporal ni en el porcentaje de grasa corporal. La calidad de los estudios varió, con heterogeneidad en el diseño y la implementación de las intervenciones, por lo que estos resultados deben interpretarse con cautela. Los hallazgos sugieren que, aunque las intervenciones en el entorno alimentario escolar pueden mejorar algunos comportamientos dietéticos y medidas de adiposidad, su impacto en la prevención de la obesidad sigue siendo inconcluso. Fortalecer las políticas y garantizar intervenciones estructuradas a largo plazo son aspectos cruciales para lograr mejoras de salud significativas y sostenibles en el ámbito escolar.

## Introduction

Childhood overweight and obesity are growing public health issues linked to metabolic issues such as increased body mass index (BMI), larger waist circumference (WC), and excess body fat ^1^ ^,^ ^2^. These factors predict lifelong cardiometabolic risks and are strongly associated with noncommunicable diseases (NCDs) ^3^. Globally, adolescents are experiencing increasing disability and mortality rates due to NCDs ^4^.

In response, the World Health Organization (WHO) and international governments recommend school interventions to modify dietary patterns and reduce exposure to weight-promoting factors ^5^ ^,^ ^6^ ^,^ ^7^. Notably, school food and nutrition strategies have shifted from knowledge-based to behavior-oriented approaches, emphasizing the food environment rather than solely the individual ^8^. Studies worldwide have shown that obesogenic school environments are widespread, underscoring the need for interventions to change these settings ^9^ ^,^ ^10^ ^,^ ^11^ ^,^ ^12^ ^,^ ^13^ ^,^ ^14^ as they encourage unhealthy food choices that contribute to obesity ^15^.

Thus, multi-component interventions that promote physical activity, reduce sedentary behavior, and improve food environments and eating habits are more effective in integrating environmental factors with individual actions ^16^ ^,^ ^17^ ^,^ ^18^ ^,^ ^19^ ^,^ ^20^ ^,^ ^21^. This approach shows promise in improving adiposity indicators ^18^ ^,^ ^21^, enhancing dietary habits ^17^ ^,^ ^20^, and preventing unhealthy weight gain or obesity ^17^ ^,^ ^19^. Successful strategies also require involving family members ^19^, the school community, and nutrition and health experts ^21^.

Previous systematic reviews have evaluated the impact of school food environment interventions on adiposity ^22^ ^,^ ^23^ ^,^ ^24^, metabolic parameters ^23^, or food consumption ^23^ ^,^ ^24^ ^,^ ^25^ in children and adolescents. However, evidence on their effectiveness remains inconclusive, as most reviews focus on anthropometric outcomes ^22^ ^,^ ^23^ ^,^ ^24^ or food consumption ^24^ ^,^ ^25^. Therefore, this review aims to evaluate the impact of multi-component interventions - including changes in the school food environment - on adiposity and food consumption in children and adolescents, hypothesizing that such interventions can influence both outcomes.

## Materials and methods

This systematic review of interventional studies investigates food consumption and adiposity in children and adolescents after interventions in their school food environment. The review was reported according to the *Preferred Reporting Items for Systematic Reviews and Meta-Analyses* (PRISMA) ^26^ (Supplementary Material - Table S1; https://cadernos.ensp.fiocruz.br/static//arquivo/suppl-e00152824_7623.pdf) and conducted based on the *Cochrane Handbook for Systematic Reviews of Interventions* ^27^. The protocol was registered in the International Prospective Register of Systematic Reviews (PROSPERO; CRD 42020186070).

### Search strategy

The MEDLINE (via PubMed), SciELO, CENTRAL, Scopus, Embase, Web of Science, and Clinical Trials databases were searched. Reference lists of selected articles and previous systematic reviews were also screened, and relevant cited references were included. No restrictions were applied regarding language or year of publication. The search words used were: “schools”, “child”, “adolescent”, “school canteen”, “food environment”, “environment intervention”, “nutrition intervention”, and “nutrition policy”. The search strategy was developed and performed in each database in November 2023 (Supplementary Material - Table S2; https://cadernos.ensp.fiocruz.br/static//arquivo/suppl-e00152824_7623.pdf).

### Eligibility criteria and outcomes of interest

Articles were evaluated using the Population, Intervention/Exposure, Comparison, Outcome, and Study Type (PICOS) framework (Supplementary Material - Table S3; https://cadernos.ensp.fiocruz.br/static//arquivo/suppl-e00152824_7623.pdf). Inclusion criteria comprised: (1) Students - children (> 2 years old) and adolescents (< 19 years old); (2) School food environment (internal environment and surroundings) - economic factors, nutritional aspects, school ambiance, legislation, and regulations for food sales in school facilities; (3) Adiposity - BMI, body fat percentage (%BF), WC as well as changes in food consumption (dietary intake); (4) Cluster randomized controlled trials (cRCTs), quasi-experimental (QE) studies, and field trials (FTs).

Exclusion criteria included observational studies, studies with mixed populations (including adults or older adults), studies based solely on educational interventions rather than the food environment, studies reporting only food consumption outcomes, systematic reviews and meta-analyses, letters, editorials, and articles repeating information from previously included populations.

### Study selection, data collection process, and data items

Based on the inclusion and exclusion criteria, three reviewers (L.A.V., B.C.C. and T.P.R.S.) screened duplicate titles and abstracts using the reference management software Rayyan (https://www.rayyan.ai/). Full-text articles were assessed separately by two investigators (L.A.V. and B.C.C.) for eligibility; disagreements were resolved by consensus or, if necessary, by consulting a third reviewer (L.L.R.). For abstracts from scientific meetings and symposia that met the criteria, authors were contacted for detailed information about recent publications or presented data. Data were extracted independently by two reviewers, duplicated, and organized in an Excel spreadsheet (https://products.office.com/), which included general study characteristics (title, authors, publication year, location), methods (design, measures of effect), participant characteristics (school grade, intervention components), outcomes, and main results. A pilot test of the data collection form was conducted, and all reviewers were trained before and during the survey.

### Statistical analysis

Meta-analyses were conducted using the DerSimonian and Laird random-effects model. This approach was chosen because it yields more conservative estimates and accounts for potential unobserved heterogeneity across studies, providing a more robust synthesis of the evidence.

Meta-analyses were presented and interpreted separately based on the study design, as recommended by the *Cochrane Handbook for Systematic Reviews of Interventions* (section 23.2.6) ^27^. Treatment effects for continuous outcomes were expressed as mean differences (MD) with 95% confidence intervals (95%CI). When available, the difference between final and baseline values was used for analysis. Forest plots were generated to present the meta-analysis results.

Heterogeneity among studies was assessed using Cochran’s Q test, with p-values < 0.10 considered statistically significant. The I^2^ test was used to evaluate the magnitude of heterogeneity, classified as moderate when I^2^ > 25% and high when I^2^ > 75%. Analyses were performed in R Statistical Software, version 4.4.0 (http://www.r-project.org), using the *Meta* (version 6.2.1) ^28^ and *Metafor* (version 4.3.0) ^29^ packages. No dichotomous outcomes were reported in primary studies; only continuous outcomes were analyzed. Sensitivity analysis was performed by excluding studies with high or serious risk of bias, and results are presented in the supplementary material. Publication bias was assessed via visual inspection of funnel plots and Egger’s test when a meta-analysis included ten or more studies. If publication bias was detected, it was corrected using the Trim-and-Fill method, and the impact of the correction on result interpretation was evaluated ^27^.

### Risk of bias within and across studies

The methodological quality of the primary studies was evaluated using the revised risk of bias (ROB 2.0) for randomized controlled trials. For QE studies, risk of bias was assessed using the non-randomized intervention studies tool (ROBINS-I), following Cochrane Collaboration recommendations ^27^. Each study assessed with ROB 2.0 was evaluated across five domains: (1) bias arising from the randomization process; (2) bias due to deviations from intended interventions; (3) bias due to missing outcome data; (4) bias in the outcome measurement; and (5) bias in the selection of the reported result. Risk of bias judgments were classified as (a) low risk, (b) some concerns, or (c) a high risk. If any specific domain was rated as higher risk, the overall risk of bias assigned to the study was assigned at least at the same level of severity.

The ROBINS-I tool is based on seven domains, namely: (1) bias due to confounding; (2) bias due to participant selection; (3) bias in classification of interventions; (4) bias due to deviations from intended interventions; (5) bias due to missing data; (6) bias in outcome measurement; and (7) bias in selection of the reported result. Risk of bias was classified as (a) low risk, (b) moderate risk, (c) serious risk, (d) critical risk, or (e) no information. We considered age and sex as the minimum set of confounding variables. Studies that did not adjust for these variables were rated as at least moderate risk of bias due to confounding.

The overall certainty of evidence for each outcome was evaluated based on the *Grading of Recommendations Assessment, Development, and Evaluation* (GRADE) system ^30^.

## Results

**Figure 1:**
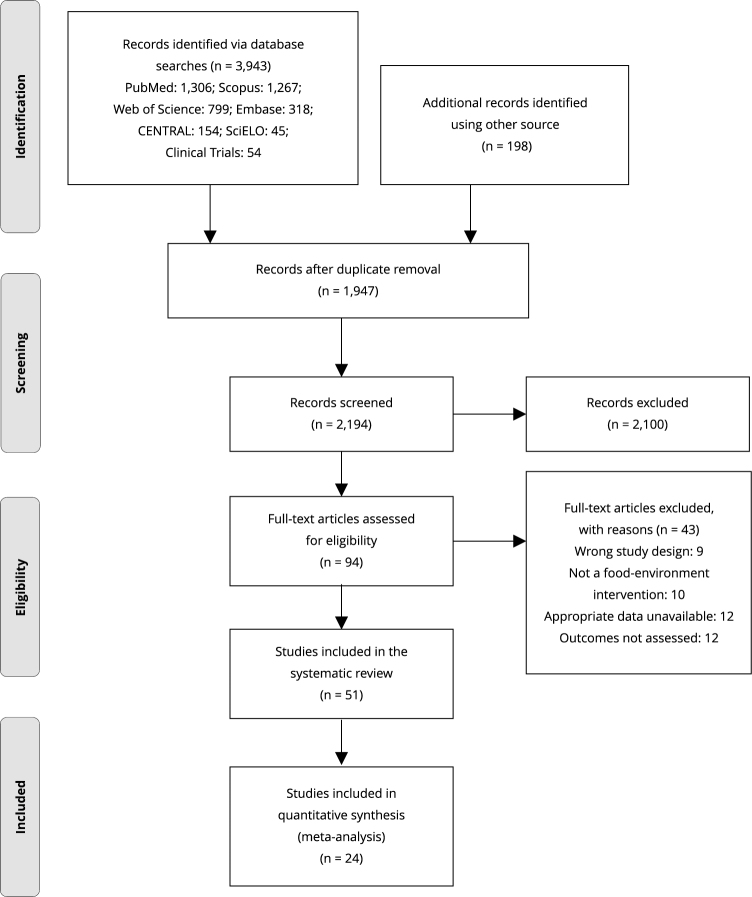
Flowchart for identifying and selecting eligible studies for the systematic review.

The search strategy identified 4,141 records (1,306 in PubMed, 1,267 in Scopus, 799 in Web of Science, 318 in Embase, 154 in CENTRAL, 45 in SciELO, and 198 in other sources). After removing duplicates (n = 1,947) and screening titles and abstracts (n = 2,194), 95 full-text records were assessed for eligibility. A total of 51 publications were included in this review, and 24 were included in the quantitative synthesis. A flowchart showing the study selection process is presented in Figure 1, and reasons for excluding studies in the second screening phase are detailed in Supplementary Material (Table S4; https://cadernos.ensp.fiocruz.br/static//arquivo/suppl-e00152824_7623.pdf).

### Study characteristics

Study characteristics and outcomes are presented in Table 1 ^31^ ^,^ ^32^ ^,^ ^33^ ^,^ ^34^ ^,^ ^35^ ^,^ ^36^ ^,^ ^37^ ^,^ ^38^ ^,^ ^39^ ^,^ ^40^ ^,^ ^41^ ^,^ ^42^ ^,^ ^43^ ^,^ ^44^ ^,^ ^45^ ^,^ ^46^ ^,^ ^47^ ^,^ ^48^ ^,^ ^49^ ^,^ ^50^ ^,^ ^51^ ^,^ ^52^ ^,^ ^53^ ^,^ ^54^ ^,^ ^55^ ^,^ ^56^ ^,^ ^57^ ^,^ ^58^ ^,^ ^59^ ^,^ ^60^ ^,^ ^61^ ^,^ ^62^ ^,^ ^63^ ^,^ ^64^ ^,^ ^65^ ^,^ ^66^ ^,^ ^67^ ^,^ ^68^ ^,^ ^69^ ^,^ ^70^ ^,^ ^71^ ^,^ ^72^ ^,^ ^73^ ^,^ ^74^ ^,^ ^75^ ^,^ ^76^ ^,^ ^77^ ^,^ ^78^ ^,^ ^79^ ^,^ ^80^ ^,^ ^81^. The earliest publication dates from 1996, and 76.5% (n = 39) of the selected articles were published between 2009 and 2023. The most frequent study designs were cRCT (n = 39; 76.5%) ^31^ ^,^ ^32^ ^,^ ^33^ ^,^ ^34^ ^,^ ^35^ ^,^ ^36^ ^,^ ^37^ ^,^ ^38^ ^,^ ^39^ ^,^ ^40^ ^,^ ^41^ ^,^ ^42^ ^,^ ^43^ ^,^ ^44^ ^,^ ^45^ ^,^ ^46^ ^,^ ^47^ ^,^ ^49^ ^,^ ^50^ ^,^ ^57^ ^,^ ^58^ ^,^ ^59^ ^,^ ^60^ ^,^ ^61^ ^,^ ^63^ ^,^ ^65^ ^,^ ^66^ ^,^ ^67^ ^,^ ^68^ ^,^ ^69^ ^,^ ^70^ ^,^ ^71^ ^,^ ^72^ ^,^ ^73^ ^,^ ^76^ ^,^ ^77^ ^,^ ^78^ ^,^ ^79^ ^,^ ^80^ ^,^ ^81^, followed by QE (n = 12; 23.5%) ^43^ ^,^ ^48^ ^,^ ^50^ ^,^ ^51^ ^,^ ^52^ ^,^ ^53^ ^,^ ^54^ ^,^ ^56^ ^,^ ^62^ ^,^ ^64^ ^,^ ^74^ ^,^ ^75^. Trials were conducted in North America (n = 21; 41.2%) ^55^ ^,^ ^56^ ^,^ ^57^ ^,^ ^58^ ^,^ ^59^ ^,^ ^66^ ^,^ ^67^ ^,^ ^68^ ^,^ ^69^ ^,^ ^70^ ^,^ ^71^ ^,^ ^72^ ^,^ ^73^ ^,^ ^74^ ^,^ ^75^ ^,^ ^76^ ^,^ ^77^ ^,^ ^78^ ^,^ ^79^ ^,^ ^80^ ^,^ ^81^, Asia (n = 15; 29.4%) ^36^ ^,^ ^37^ ^,^ ^38^ ^,^ ^39^ ^,^ ^40^ ^,^ ^46^ ^,^ ^47^ ^,^ ^48^ ^,^ ^49^ ^,^ ^51^ ^,^ ^52^ ^,^ ^53^ ^,^ ^54^ ^,^ ^64^ ^,^ ^65^, Europe (n = 8; 15.7%) ^34^ ^,^ ^42^ ^,^ ^44^ ^,^ ^45^ ^,^ ^50^ ^,^ ^60^ ^,^ ^61^ ^,^ ^63^, Oceania (n = 3; 5.9%) ^32^ ^,^ ^33^ ^,^ ^43^, and Latin America (n = 4; 7.8%) ^31^ ^,^ ^35^ ^,^ ^41^ ^,^ ^62^. Most populations consisted of primary school students (n = 35; 68.6%) ^31^ ^,^ ^32^ ^,^ ^35^ ^,^ ^36^ ^,^ ^37^ ^,^ ^38^ ^,^ ^39^ ^,^ ^40^ ^,^ ^42^ ^,^ ^44^ ^,^ ^45^ ^,^ ^48^ ^,^ ^49^ ^,^ ^51^ ^,^ ^52^ ^,^ ^53^ ^,^ ^55^ ^,^ ^56^ ^,^ ^57^ ^,^ ^58^ ^,^ ^59^ ^,^ ^62^ ^,^ ^63^ ^,^ ^64^ ^,^ ^66^ ^,^ ^67^ ^,^ ^68^ ^,^ ^70^ ^,^ ^71^ ^,^ ^73^ ^,^ ^74^ ^,^ ^75^ ^,^ ^79^ ^,^ ^80^ ^,^ ^81^, secondary school students (n = 12; 23.5%) ^33^ ^,^ ^34^ ^,^ ^41^ ^,^ ^43^ ^,^ ^46^ ^,^ ^47^ ^,^ ^50^ ^,^ ^54^ ^,^ ^60^ ^,^ ^61^ ^,^ ^69^ ^,^ ^76^, or both (n = 4; 7.8%) ^65^ ^,^ ^72^ ^,^ ^77^ ^,^ ^78^. The median intervention duration was 10 months (range 1-48). Most studies (n = 41; 80.4%) ^31^ ^,^ ^32^ ^,^ ^33^ ^,^ ^34^ ^,^ ^35^ ^,^ ^37^ ^,^ ^39^ ^,^ ^40^ ^,^ ^41^ ^,^ ^42^ ^,^ ^43^ ^,^ ^44^ ^,^ ^45^ ^,^ ^46^ ^,^ ^47^ ^,^ ^49^ ^,^ ^50^ ^,^ ^51^ ^,^ ^53^ ^,^ ^54^ ^,^ ^55^ ^,^ ^57^ ^,^ ^58^ ^,^ ^59^ ^,^ ^62^ ^,^ ^63^ ^,^ ^64^ ^,^ ^65^ ^,^ ^66^ ^,^ ^67^ ^,^ ^69^ ^,^ ^70^ ^,^ ^72^ ^,^ ^73^ ^,^ ^74^ ^,^ ^75^ ^,^ ^76^ ^,^ ^77^ ^,^ ^78^ ^,^ ^80^ ^,^ ^81^ assessed outcomes immediately after the intervention, with no follow-up. Among studies that conducted follow-up, the median duration was six months (range 1-36).

**Table 1:** Summary of study characteristics, school-based interventions, and statistically significant outcomes (1996-2023, n = 51).

Country/Year (study)	Study design/ Intervention duration	Sample: schools (n)/students (n total/CG/IG) */age range	Intervention components		Main results **
			Environmental	Individual	
Argentina/2013 (Herscovici et al. ^31^)	cRCT 6 months	6 405 (171/234) 9-11 years	- Offering healthier foods	- Workshops about healthy eating habits and physical activity with students - Health-focused workshops with parents at school	*Compared to CG:* - boys and girls: ↓ consumption of hamburgers and hot dogs - girls: ↑ consumption of skim milk and orange juice
Australia/2014 (Chellappah et al. ^32^)	cRCT 8 weeks	4 271 (137/134) 9-10 years	- Offering fruits every day during break - Encouraging students to eat fruits in class before break	- Did not include this component	*Compared to baseline:* - ↓ WC *Compared to CG:* - ↑ vegetable and fruit intake
Australia/2021 (Ooi et al. ^33^)	cRCT 6 months	6 862 (389/473) 12-15 years	- Decreasing accessibility and appeal of SSB - Changing the regular curriculum - Installing water fountains - Peer-led school challenge designed and led by a student committee	- Lessons about SSB consumption with students - Sending health messages (push notifications) to students and parents - Sending newsletter snippets to parents	No significant differences
Belgium/2006 (Haerens et al. ^34^)	cRCT 2 years	15 2,991 (759/2,232 ***) 11-15 years	*Group 1 + Group 2:* - Offering fruit at very low prices or free of charge at least once a week - Offering water at a lower price than soft drinks or for free - Offering fruit as a dessert during lunch break - Hanging health-related folders and posters - Providing free water cans - Increasing the amount of time students spend in moderate-to-vigorous physical activity - Promoting physical activity during the school day and after school - Receiving box with sports materials (ropes, Frisbees, etc.)	*Group 1 + Group 2:* - Encouraging teachers to organize extra support activities (healthy food) - Getting personalized feedback regarding physical activity and healthy eating for students *Only Group 1:* - Sending communications and activities and involving parents in interactive meetings	*At 2 years follow-up, compared Group 1 to CG:* - girls: lower increase in BMI and BMI (z-score) *At 2 years follow-up, compared Group 1 to Group 2:* - girls: lower increase in BMI (z-score)
Chile/2004 (Kain et al. ^35^)	cRCT 6 months	5 3,086 (945/2,141) 6-10 years	- Changing the regular curriculum - Training school canteen staff (including owners) - Providing extra physical activity time for students - Providing sports equipment	- Lessons about healthy eating and physical activity - Healthy snacks contest (stickers and prize) - Training sessions with teachers - Involving parents in meetings	*Compared to baseline:* - boys: ↓ BMI (z-score), WC
China/2014 and 2015 (Xu et al. ^36^ ^,^ ^37^)	cRCT 8 months	8 1,182 (544/638) 10 years	- Changing the regular curriculum - Hanging health-related messages and posters (school canteen, classroom, gymnasium, and playground) - Promoting fun events	- Lessons about healthy eating habits and physical activity with students - Training sessions with teachers - Involving parents in health classes at school	*Compared to CG:* - ↓ consumption frequency of red meat, fried snacks and soft drinks - ↑ consumption frequency of vegetables - IG was more likely to achieve a 0.5kg BMI reduction
China/2015 (Cao et al. ^38^)	cRCT 2 years + 9 months	14 1,854 (889/965) 6-7 years	- Offering more fruits and vegetables in the school canteen - Reducing the fat content of food - Disseminating obesity-related health information (blackboard newspaper, morning meeting, and class meeting) - Increasing the amount of time students spend in physical activity - Providing a strip of skipping rope to students (physical activity at home)	- Lessons about health education (theme class meetings or seminars, brochures) to students and parents - Training sessions with teachers - Sending communications and activities to parents	*At 3 years follow-up, compared to CG:* - ↓ odds of developing obesity or overweight - ↓ BMI (z-score) (especially for overweight and obese students)
China/2019 (Liu et al. ^39^)	cRCT 1 year	12 1,889 (959/930) 7-11 years	- Changing rules regarding selling unhealthy foods - Not allowing SSB, unhealthy snacks, and electronic products (smartphones and tablets) in school - Providing practical suggestions to improve children’s dietary intake - Changing the regular curriculum - Hanging posters about health education knowledge - Increasing the amount of time students spend in moderate-to-vigorous physical activity - Providing sports equipment (rope jumping and shuttlecock kicking)	- Lessons about behavioral knowledge, skills, healthy eating habits, childhood obesity prevention, and physical activity with students - Training sessions with teachers - Encouraging extracurricular activities at home - Involving parents in extracurricular physical activity and discussions about intervention approaches	*At 12 months follow-up, compared to CG:* - ↓ percentage of children consuming SSB/day
China/2019 (Li et al. ^40^)	cRCT 1 year	40 1,641 (809/832) 6-7 years	- Providing vegetables every day - Reducing the fat, sugar, and salt content of school meals - Offering smaller portion sizes - Testing school lunch improvement goals (school staff and commercial suppliers) - Creating a committee (physical activity levels) - Challenge of healthy behaviors (students, parents, teachers) - Increasing the amount of physical activity	- Lessons and workshops about healthy eating and an active lifestyle with students - Handing out program handbooks (intervention activities) to school’s principals and class teachers - Promoting fun and active family games - Involving parents in workshops (with educational leaflets)	*Compared to CG:* - ↓ BMI z-score, WC - ↑ consuming at least five daily portions of fruit and vegetables - ↓ weekly consumption of SSB and unhealthy snacks
Ecuador/2017 (Ochoa-Avilés et al. ^41^)	cRCT 2 years + 4 months	20 1,430 (728/702) 12-14 years	- Changing the regular curriculum - Training school staff (recipes, leaflets, workshops)	- Lessons and workshops about healthy eating habits (booklets, games, didactic material) with students - Preparation of a healthy breakfast with students - Involving parents in workshops at school (booklets)	*Compared to baseline:* - ↓ WC - ↓ unhealthy snacks, added sugar, daily fruit and vegetables intake (decreased in both groups, but lower in IG)
England/2001 (Sahota et al. ^42^)	cRCT 1 year	10 613 (312/301) 7-11 years	- Offering healthier foods - Changing the regular curriculum - Additional sessions supplied by the project manager	- Lessons about healthy eating habits and physical activity with students - Training sessions with teachers	*Compared to baseline:* - ↑ vegetable intake among all children - ↓ fruit intake among obese children - ↑ consumption of high-sugar foods and drinks among overweight children
Fiji/2011 (Kremer et al. ^43^)	QE 2 years	18 7,237 (4,567/2,670) 13-18 years	- Offering healthier meals - Training school canteen staff (offering breakfast, opening earlier) - Changing the regular curriculum - Changing school policies for a healthy canteen (guidelines) - Building school gardens - Hanging posters and distributing pamphlets about healthy snacks - Promoting events, assembly (healthy eating habits) with students - Promoting physical activity during the school day - Providing sports equipment (hoops, ropes) and water bottles	- Lessons about healthy eating habits with students and parents - Training on vegetable garden, pot plant technology, healthy meal preparation - Training sessions with teachers - Sending communications (newsletter, school website) and activities to parents	*Compared to CG:* - ↓ %BF - ↑ vegetable intake (at school)
German/2009 (Muckelbauer et al. ^44^ ^,^ ^45^)	cRCT 10 months	33 3,190 (1,469/1,721) 7-8 years	- Providing water bottles for children - Installing water fountains provided cooled, filtered, plain, or optionally carbonated water	- Lessons about the importance of water for the body and the water circuit in nature with students - Training sessions with teachers	*Compared to CG:* - ↓ risk of overweight - ↑ water consumption
India/2010 (Singhal et al. ^46^)	cRCT 6 months	2 201 (102/99) 15-17 years	- Offering healthier foods - Stopping the sale of unhealthy foods - Promoting physical activity during the school day and after school	- Lessons about healthy eating habits with students - Conducting a health camp with teachers and parents (nutritional counseling) - Counseling by phone for parents - Individual counseling by nutritionist	*Compared to CG:* - ↓ WC - ↓ proportion of children consumed aerated drinks, unhealthy foods (burger/pizza/French fries/noodles) - ↑ proportion of children brought tiffin (packed lunch) and brought a fruit in their tiffin
India/2011 (Singhal et al. ^47^)	cRCT 6 months	2 134 (57/77) 15-17 years	- Offering healthier foods - Stopping the sale of unhealthy foods - Promoting physical activity during the school day and after school	- Lessons about healthy eating habits with students - Conducting a health camp with teachers and parents (nutritional counseling) - Counseling by phone for parents - Individual counseling with children held by a nutritionist	*Compared to CG:* - ↓ WC
Indonesia/2022 (Kurniawan et al. ^48^)	QE 5 months	2 350 (196/164) 9-11 years	- Training canteen staff - Training students’ peer leader club	- Lessons about eating habits and physical activity with students - Promoting healthy home food weekly to be eaten together at school during recess - Training sessions with teachers - Involving parents in seminars (health promotion) - Sending communications (leaflets) to parents	*Compared to baseline:* ↑ BMI (both groups, but higher in CG) *Compared to CG:* - ↑ eating fruits and vegetables behavior
Iran/2016 (Amini et al. ^49^)	cRCT 18 weeks	12 334 ^#^ (167/167) 10-12 years	- Offering healthier foods - Stopping the sale of unhealthy foods - Increasing the amount of physical activity	- Lessons about healthy eating habits with students - Involving parents in health classes at school	*Compared to CG:* - ↓ BMI (z-score) *Compared to baseline:* - ↑ WC (both groups, but higher in CG) - ↑ energy, fat intake
Italia/2016 (Ermetici et al. ^50^)	QE 2 years	6 487 (225/262) 11-15 years	- Replacing unhealthy foods with healthy foods and beverages in vending machines - Changing the regular curriculum - Hanging posters (healthy diet, water consumption, physical activity) - Providing a reusable water bottle - Creating more opportunities for exercise during breaks (an additional hour a week of movement) - Giving a pedometer to students	- Lessons about healthy eating habits (textbook) with students - Sending communications (automated text messages) to parents and students - Involving parents in activities sent home (textbook)	*Compared to CG:* - ↓ BMI (z-score) - ↓ SSB, high-energy snack consumption *Intervention effect in subgroup analysis:* - ↓ BMI (z-score) and high-energy snacks consumption in girls with overweight/obesity
Lebanon/2014 (Habib-Mourad et al. ^51^)	cRCT 3 months	8 374 (181/193) 9-11 years	- Changing the regular curriculum - Training canteen staff (recommendations about healthy snacks and drinks) - Hanging posters (healthy food choices) in the school canteen	- Lessons about healthy eating habits and physical activity (fun and interactive activities) with students - Training sessions with teachers - Involving parents in meetings, school events, and activities sent home (food samples, recipes, healthy lunch boxes) - Sending communications to parents (pamphlets)	No significant differences
Malaysia/2018 (Koo et al. ^52^)	QE 3 months	2 83 ^#^ (40/43) 9-11 years	- Changing availability of whole grain foods (delivered daily in school)	- Lessons about whole grains recommendations and a balanced diet - Individual diet counseling for parents (booklet and recipes with whole grain foods)	*At 9 months follow-up, compared to CG:* - ↓ BMI-for-age (z-score), %BF, WC *At 3 months follow-up, compared to baseline:* - ↓ %BF, WC
Malaysia/2021 (Teo et al. ^53^)	QE 3 months	6 523 (272/251) 7-11 years	- Offering healthier foods - Training school canteen staff - Providing a skipping rope to students	- Lessons about health awareness, nutrition, food hygiene, and physical activity with students - Training sessions with teachers - Involving parents in monitoring the program’s implementation (visit the school canteen)	*At 3 months follow-up, compared to CG:* - ↓ BMI-for-age
Malaysia/2022 (Majid et al. ^54^)	QE 4 weeks	6 340 ^##^ (93/247 ***) 14-15 years	*Intervention one + two:* - Training school canteen staff - Giving a “Healthy Canteen Booklet” to school canteen staff *Only intervention two:* - Subsidy for healthy foods (fruits, vegetables, low-energy-dense *kuih* - traditional cake) - Students receive coupons that subsidize the price of healthy foods weekly - Allocation of funds to prepare healthy food was given to the school - Installing water fountains	- Did not include this component	*Compared to baseline:* - ↓ WC in intervention two - ↓ energy in all arms - ↑ fat intake and %BF in intervention one
Mexico/2009 (Colín-Ramirez et al. ^55^)	cRCT 1 year	10 619 (315/304) 8-10 years	- Offering healthier foods - Creating more opportunities for exercise during the school day	- Lessons about healthy eating habits and physical activity (classes and fun activities) with students - Training sessions with teachers - Involving parents in lectures and activities at home (menu and snack suggestions, recommendations for a healthy lifestyle)	*Compared to baseline:* - ↓ energy and saturated fat intakes - ↑ WC in both groups (only significant in CG)
Mexico/2012 (Bacardí-Gascon et al. ^56^)	QE 6 months	4 532 (280 ^###^/252) 8-9 years	- Offering healthier foods - Changing the regular curriculum	- Lessons about healthy eating habits and physical activity with students and parents - Meetings with the school board and teachers (improve school meals, snacks offered in the school canteen, and physical activity installations)	*At 6 months follow-up, compared to CG:* - ↓ BMI *At 24 months follow-up, compared pre- to post-intervention:* - ↑ BMI, BMI (z-score), WC - ↓ abdominal obesity (WC > 90th percentile) - ↑ vegetables intake, SSB - ↓ consumption of snacks high in fat and salt, availability of SSB, cookies, chocolates, candy, and vegetables at home
Mexico/2012 (Levy et al. ^57^)	cRCT 6 months	60 1,019 (510/509) 10 years	- Offering healthier foods - Training school canteen staff - Hanging posters (healthy breaks) - Broadcasting of audio spots on the schools’ physical activity systems to promote the consumption of water, fruits, vegetables, and physical activity - Hanging posters (physical activity) - Providing a school guide (to support physical activity), a CD with music for established activities, and a bottle of water for the children - Organizing games during the break	- Lessons about healthy eating habits and physical activity (workshops, puppet theatre) with students - Training sessions with teachers - Participation of teachers in games during the break - Teachers performing activities before the start of classes (warm-ups, activation and relaxation) - Delivery of recipe calendars (healthy eating and physical activity) to parents	*Compared to baseline:* - ↓ probability of shifting from the overweight to the obesity category
Mexico/2013 (Alvirde-García et al. ^58^)	cRCT 3 years	5 2,682 (755/1,927) 9 years	- Offering healthier foods - Meetings with school canteen staff - Changing the regular curriculum - Increasing the amount of time students spend in moderate-to-vigorous physical activity - Promoting physical activity during and after school (guide and activity cards)	- Lessons about healthy eating habits and physical activity (textbook and workbook) with students and parents - Training sessions with teachers - Involving parents in meetings and activities at home	*At 3 years follow-up, compared to CG:* - ↓ increase of BMI variation - ↓ energy, bread, fat and sugar intake *At 3 years follow-up, compared to baseline:* - ↓ energy in both groups (higher in IG) *At 3 years follow-up, compared to 1 year follow-up:* - ↑ BMI in both groups
Mexico/2013 (Safdie et al. ^59^)	cRCT 1.5 years	27 860 (354/506 ***) 9-11 years	*Group 1 + Group 2:* - Increasing availability of healthy foods and water at school - Reducing availability of unhealthy foods - Training school canteen staff and school authorities (booklets, workshops) - Providing sports equipment - Promoting physical activity during the school day *Only Group 2:* - Increasing the amount of time students spend in moderate-to-vigorous physical activity - Additional financial investment and human resources (hiring physical education teachers)	*Group 1 + Group 2:* - Promoting workshops, mass communication, and marketing strategies targeted to students (healthy eating habits) - Training sessions with teachers - Distributing a booklet for parents (healthy lunch)	*At 18 months follow-up, compared to CG:* - ↓ consumption of unhealthy foods at school (only group 2) *At 18 months follow-up, compared to baseline:* - ↑ availability of healthy foods at school (both IG) - ↓ availability of unhealthy foods at school (both IG)
Netherlands/2007 and 2009 (Singh et al. ^60^ ^,^ ^61^)	cRCT 8 months	18 1,053 (453/600) 12-13 years	- Offering smaller portion sizes - Offering healthier foods - Changing the regular curriculum - Restricting access to vending machines - Providing financial encouragement to schools to offer additional physical activity options	- Biology and physical activity lessons with students - Training sessions with teachers	*At 8 months follow-up, compared to CG:* - girls: ↓ SSB consumption - boys: ↓ WC, SSB consumption *At 12 months follow-up, compared to CG:* - girls: ↓ SSB consumption - boys: ↓ SSB consumption
Peru/2017 (Aparco et al. ^62^)	QE 9 months	4 824 (347/477) 6-10 years	- Training school canteen staff - Promoting physical activity during the school recess - Providing sports equipment (balls, cones, ropes)	- Lessons about healthy eating habits and physical activity (educational materials, puppet theatre) with students - Training sessions with teachers - Involving parents in lessons about healthy eating habits and physical activity and activities at home	*Compared to baseline:* - ↓ vegetable consumption
Sweden/2009 (Marcus et al. ^63^)	cRCT 4 years	10 3,135 (1,465/1,670) 6-10 years	- Offering healthier foods - Changing the regular curriculum - Increasing the amount of physical activity - Increasing opportunities for physical activity	- Training sessions with teachers - Communicating with parents	*Compared to baseline:* - Eating habits at home were healthier (↓ sweetened cereals, high-fat dairy, and sweet products)
Thailand/2017 (Chawla et al. ^64^)	QE 6 months	4 453 (227/226) 8-12 years	- Offering healthier foods - Stopping the sale of unhealthy foods - Removing vending machines - Training sessions with school staff (comic book, discussions, handbook) and teachers - Requesting vendors around the school to stop selling unhealthy food	- Lessons about healthy eating habits, obesity consequences, growing vegetables, and physical activity (presentations, gaming, gardening/planting, comic book) with students - Involving parents in workshops, activities sent home (healthy handbook), and sending communications (report cards, meetings)	*Compared to CG:* - ↓ sugary foods (chocolate and candies), fast foods consumption - ↑ vegetables consumption
Turkey/2011 (Sevinç et al. ^65^)	cRCT 8 months	6 6,847 (2,926/3,921 ***) 7-13 years	*Group 1 + Group 2:* - Offering healthier foods in the school canteen - Distributing boxed milk during mealtime	*Group 1 + Group 2:* - Lessons about healthy eating and methods of preventing obesity *Only in Group 1:* - Lessons about physical activity	*Group 1 and Group 2, compared to CG:* - ↑ BMI (lower than CG increased) - ↓ BMI increased in boys IG compared to CG *Group 1, compared to CG and group 2:* - ↓ BMI increased in boys compared to girls
United States/1996 and 1999 (Luepker et al. ^66^; Webber et al. ^67^; Nader et al. ^68^)	cRCT 3 years	96 4,019 ^#^ (1,653/2,366 **) 8-11 years	*Group 1 + Group 2:* - Training school canteen staff - Changing the regular curriculum - Increasing the amount of time students spend in moderate-to-vigorous physical activity	*Group 1 + Group 2:* - Lessons about healthy eating habits, cigarette smoking, and physical activity with students - Training sessions with teachers and physical education specialists *Only in Group 1:* - Involving parents in activities sent home and in school events	*At 3 years follow-up, Group 1 + Group 2, compared to CG:* - ↓ % energy from total fat, saturated fat - ↑ total daily energy intake (both groups, but lower in IG) *At 6 years follow-up, Group 1 + Group 2, compared to CG:* - ↓ % energy from total fat and saturated fat intake
United States/2003 (Sallis et al. ^69^)	cRCT 2 years	24 1,678 (no data) 11-13 years	- Offering healthier foods - Providing financial encouragement to purchase kitchen and physical activity equipment - Health policy meetings - Creating a committee - Promoting physical activity before and after school, as well as after lunch	- Training sessions with teachers - Sending communications and involving parents in school events and meetings	*Compared to CG:* - ↓ BMI only in boys
United States/2003 (Caballero et al. ^70^)	cRCT 3 years	41 1,704 (825/879) 8-10 years	- Reducing the fat content of school meals - Training school canteen staff - Changing the regular curriculum - Increasing the amount of time students spend in moderate-to-vigorous physical activity - Promoting physical activity in the classroom	- Lessons about healthy eating habits and physical activity with students - Training sessions with teachers - Communicating with and involving parents in school events	*Compared to CG:* - ↓ % energy from fat (24-hR, and direct observation), total energy intake (only 24-hR, not by direct observation)
United States/2004 (Treviño et al. ^71^)	cRCT 7 months	27 1,419 (706/713) 9 years	- Changing the regular curriculum - Training school canteen staff	- Lessons about healthy eating habits and physical activity with students - Training sessions with teachers - Involving parents in events, meetings, and activities at home	No significant differences
United States/2007 (Williamson et al. ^72^)	cRCT 1.5 year	4 661 (313/348 ^§^) 7-11 years	- Offering healthier foods - Eliminating vending machines - Reducing unhealthy foods - Training school canteen staff - Creating marketing materials (menu boards, buttons, place cards) and a committee - Hanging posters (physical activity) - Promoting physical activity during and after school - Providing financial encouragement to purchase physical activity equipment	- Training sessions with teachers - Sending communications, creating a website, and involving parents in committee	*Compared to CG:* - ↓ total calories, % of calories from total dietary fat, saturated fat
United States/2008 (Foster et al. ^73^)	cRCT 2 years	10 1,349 (600/749) 9-11 years	- Offering healthier foods - Creating a committee - Training all school staff	- Lessons about healthy eating habits and physical activity with students - Involving parents in committees, meetings, and nutrition workshops	No significant differences
United States/2010 (Hollar et al. ^74^)	QE 2 years	5 3,769 (737/3,032) 6-13 years	- Offering healthier foods - Reducing unhealthy foods - Changing the regular curriculum - Hanging health-related posters - Providing a school gardening program - Increasing opportunities for physical activity	- Lessons about healthy eating habits and physical activity with students - Training sessions with teachers - Sending communications to parents (newsletter)	*At 2 years follow-up, compared to CG:* - ↓ BMI (percentile)
United States/2010 (Hollar et al. ^75^)	QE 2 years	5 2,494 (465/2,029) 6-13 years	- Offering healthier foods - Reducing unhealthy foods - Changing the regular curriculum - Hanging health-related posters - Providing a school gardening program - Increasing opportunities for physical activity	- Lessons about healthy eating habits and physical activity with students - Training sessions with teachers - Sending newsletters to parents	*At 2 years follow-up, compared to CG:* - girls: ↓ BMI (z-score)
United States/2010 (Foster et al. ^76^)	cRCT 3 years	42 6,358 (3,169/3,189) 11 years	- Offering healthier foods - Reducing unhealthy foods - Increasing the amount of time students spend in moderate-to-vigorous physical activity - Providing financial encouragement to purchase physical activity equipment	- Lessons about behavioral knowledge and skills with students - Training sessions with physical education teachers - Involving parents in activities sent home and sending communications	*Compared to CG:* - ↓ BMI (z-score), % of students with WC (≥ percentile 90), % of students with BMI ≥ percentile 85 (both groups)
United States/2012 (Coleman et al. ^77^)	cRCT 2 years	8 579 (279/300) 7-11 years	- Offering healthier foods - Reducing unhealthy foods - Training school canteen staff - Hanging health-related posters - Performing taste tests on students - School staff modeling healthy eating - Providing free meals for staff who eat school lunches with students	- Lessons about healthy eating habits with students - Participation of teachers and parents in the development of action plans to change school environment - Meetings with parents	*Compared to baseline:* - ↑ BMI (z-score) (both groups)
United States/2012 (Williamson et al. ^78^)	cRCT 2 years + 4 months	17 2,060 (587/1,473 ***) 9-11 years	*Group 1 + Group 2:* - Offering healthier foods (vending machine and school canteen) - Reducing unhealthy foods - Training school canteen staff - Creating marketing materials (menu boards, buttons, place cards) - Creating a committee - Hanging posters (physical activity) - Promoting physical activity during the school day and after school - Providing financial encouragement to purchase physical activity equipment	*Group 1 + Group 2:* - Training sessions with teachers - Sending communications, creating a website, and involving parents in committee *Only Group 2:* - Lessons about healthy eating habits with students - Internet counseling for children and their parents	*At 28 months follow-up, compared Group 1 + Group 2 to CG:* - ↓ %BF in boys
United States/2016 (Bogart et al. ^79^)	cRCT 5 weeks	10 2,439 (1,261/1,178) 12-13 years	- Offering healthier foods - Offering a greater variety of sliced/bite-sized food and freely chilled filtered water at lunch - Training with students’ peer leader club (social marketing: intervention messages at lunchtime, taste tests, short film) - Hanging health-related and physical activity posters and nutritional postings	- Lessons about healthy eating habits and physical activity with students - Sending parent-student activities - Communicating with parents	*Compared to CG:* - ↓ BMI (percentiles) in students who were classified as obese at baseline
United States/2021 (Davis et al. ^80^)	cRCT 9 months	16 3,135 (1,723/1,412) 8-10 years	- Changing the regular curriculum - Creating a committee - Building gardens	- Lessons about nutrition, gardening, and cooking with students - Involving parents in health classes at school	*Compared to CG:* - ↑ vegetable intake
United States/2023 (Patel et al. ^81^)	cRCT 15 months	18 1,249 (572/677) 9 years	- Installing water fountains in which cups were also available - Kickoff assembly - Signage and modest prizes for students observed drinking water at lunch	- Lessons about health, fiscal, and environmental benefits of drinking water with students - Sending parent-student activities	No significant differences

24-hR: 24-hour dietary recall; %BF: body fat percentage; BMI: body mass index; CG: control group; cRCT: cluster randomized controlled trial; IG: intervention group; QE: quasi-experimental study; SSB: sugar-sweetened beverage; WC: waist circumference.

* Number of individuals assessed at baseline was taken into consideration;

** Only outcomes with statistically significant results (p < 0.05) are reported in the “Main results” column;

*** Study with two intervention arms;

^#^ Only students with overweight or obesity were included in the study;

^##^ Included the number of students analyzed at the end of the intervention (no base data);

^###^ After six months of intervention, the control group received the intervention, and the data were analyzed as pre- and post-intervention;

^§^ The study compared two interventions (no control group).

A total of 44 interventions were found across 51 studies, each including two or more components with at least one environmental component. A description of the multi-component interventions (environmental and individual) is shown in Supplementary Material (Figure S1; https://cadernos.ensp.fiocruz.br/static//arquivo/suppl-e00152824_7623.pdf). Most interventions had environmental components targeting changes to the school canteen (n = 40; 90.9%) ^31^ ^,^ ^32^ ^,^ ^33^ ^,^ ^34^ ^,^ ^35^ ^,^ ^38^ ^,^ ^39^ ^,^ ^40^ ^,^ ^41^ ^,^ ^42^ ^,^ ^43^ ^,^ ^46^ ^,^ ^48^ ^,^ ^49^ ^,^ ^51^ ^,^ ^52^ ^,^ ^53^ ^,^ ^54^ ^,^ ^55^ ^,^ ^56^ ^,^ ^57^ ^,^ ^58^ ^,^ ^59^ ^,^ ^60^ ^,^ ^62^ ^,^ ^63^ ^,^ ^64^ ^,^ ^65^ ^,^ ^66^ ^,^ ^69^ ^,^ ^70^ ^,^ ^71^ ^,^ ^72^ ^,^ ^73^ ^,^ ^74^ ^,^ ^76^ ^,^ ^77^ ^,^ ^78^ ^,^ ^79^ ^,^ ^80^ and school policies (n = 33; 75%) ^32^ ^,^ ^33^ ^,^ ^34^ ^,^ ^35^ ^,^ ^36^ ^,^ ^38^ ^,^ ^39^ ^,^ ^40^ ^,^ ^41^ ^,^ ^42^ ^,^ ^43^ ^,^ ^44^ ^,^ ^48^ ^,^ ^50^ ^,^ ^51^ ^,^ ^54^ ^,^ ^56^ ^,^ ^57^ ^,^ ^58^ ^,^ ^60^ ^,^ ^63^ ^,^ ^64^ ^,^ ^66^ ^,^ ^69^ ^,^ ^70^ ^,^ ^72^ ^,^ ^73^ ^,^ ^74^ ^,^ ^77^ ^,^ ^78^ ^,^ ^79^ ^,^ ^80^ ^,^ ^81^. Details of school interventions and the main study results are provided in Table 1. Among individual-level components, the most common were nutrition and health education sessions (n = 38; 86.4%) ^31^ ^,^ ^33^ ^,^ ^35^ ^,^ ^36^ ^,^ ^38^ ^,^ ^39^ ^,^ ^40^ ^,^ ^41^ ^,^ ^42^ ^,^ ^43^ ^,^ ^44^ ^,^ ^46^ ^,^ ^48^ ^,^ ^49^ ^,^ ^50^ ^,^ ^51^ ^,^ ^52^ ^,^ ^53^ ^,^ ^55^ ^,^ ^56^ ^,^ ^57^ ^,^ ^58^ ^,^ ^59^ ^,^ ^60^ ^,^ ^62^ ^,^ ^64^ ^,^ ^65^ ^,^ ^66^ ^,^ ^70^ ^,^ ^71^ ^,^ ^73^ ^,^ ^74^ ^,^ ^76^ ^,^ ^77^ ^,^ ^78^ ^,^ ^79^ ^,^ ^80^ ^,^ ^81^, parent involvement (n = 36; 81.8%) ^31^ ^,^ ^33^ ^,^ ^35^ ^,^ ^36^ ^,^ ^38^ ^,^ ^39^ ^,^ ^40^ ^,^ ^41^ ^,^ ^43^ ^,^ ^46^ ^,^ ^48^ ^,^ ^49^ ^,^ ^50^ ^,^ ^51^ ^,^ ^52^ ^,^ ^53^ ^,^ ^55^ ^,^ ^57^ ^,^ ^58^ ^,^ ^59^ ^,^ ^62^ ^,^ ^63^ ^,^ ^64^ ^,^ ^66^ ^,^ ^69^ ^,^ ^70^ ^,^ ^71^ ^,^ ^72^ ^,^ ^73^ ^,^ ^74^ ^,^ ^76^ ^,^ ^77^ ^,^ ^78^ ^,^ ^79^ ^,^ ^80^ ^,^ ^81^, and teacher programs (n = 30; 68.2%) ^34^ ^,^ ^35^ ^,^ ^36^ ^,^ ^38^ ^,^ ^39^ ^,^ ^42^ ^,^ ^43^ ^,^ ^44^ ^,^ ^46^ ^,^ ^48^ ^,^ ^51^ ^,^ ^53^ ^,^ ^55^ ^,^ ^56^ ^,^ ^57^ ^,^ ^58^ ^,^ ^59^ ^,^ ^60^ ^,^ ^62^ ^,^ ^63^ ^,^ ^66^ ^,^ ^69^ ^,^ ^70^ ^,^ ^71^ ^,^ ^72^ ^,^ ^73^ ^,^ ^74^ ^,^ ^76^ ^,^ ^77^ ^,^ ^78^ (Table 1). Intervention coverage was analyzed across studies, but given their heterogeneity, it was impossible to establish any association between intervention effectiveness and duration.

The selected articles presented outcomes in two main categories: adiposity (BMI, %BF, WC) and food consumption. Figure S2 (Supplementary Material; https://cadernos.ensp.fiocruz.br/static//arquivo/suppl-e00152824_7623.pdf) summarizes the conclusions from the studies included in this review.

### Adiposity

**Figure 2:**
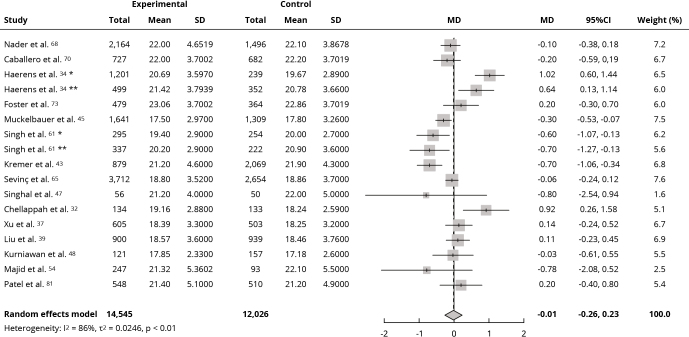
Forest plot of the effect of intervention in food environment school on body mass index (kg/m^2^).

**Figure 3:**
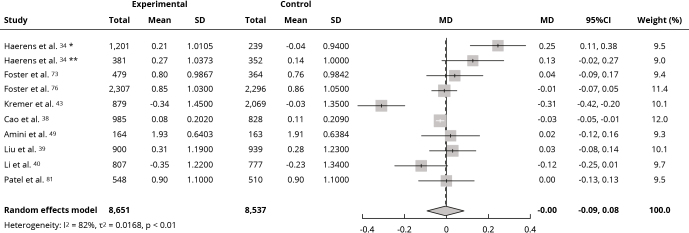
Forest plot of the effect of intervention in food environment school on body mass index (z-score).

Meta-analyses were performed using BMI data from 19 assessed studies. Interventions did not affect mean BMI (kg/m^2^) compared with the control group (MD: -0.01; 95%CI: -0.26, 0.23; I^2^ = 78%; low certainty of evidence) (Figure 2), nor mean BMI z-score (MD: -0.00; 95%CI: -0.09, 0.08; I^2^ = 82%; very low certainty of evidence) (Figure 3). Sensitivity analysis for both outcomes showed a change in effect direction after removing studies with high risk of bias, but results remained insignificant (Supplementary Material - Figures S3 and S4; https://cadernos.ensp.fiocruz.br/static//arquivo/suppl-e00152824_7623.pdf). Twenty-two studies were included in the qualitative synthesis due to incomplete data for the meta-analysis. Among these, seven ^35^ ^,^ ^50^ ^,^ ^52^ ^,^ ^53^ ^,^ ^69^ ^,^ ^74^ ^,^ ^75^ reported significant BMI reductions; in two ^35^ ^,^ ^69^, the reduction was observed only in boys, and in one ^75^ only in girls. Three studies ^56^ ^,^ ^58^ ^,^ ^77^ reported increased BMI in both groups, while the remaining studies recorded no significant changes ^31^ ^,^ ^33^ ^,^ ^42^ ^,^ ^46^ ^,^ ^51^ ^,^ ^57^ ^,^ ^59^ ^,^ ^62^ ^,^ ^72^ ^,^ ^78^ ^,^ ^79^ ^,^ ^80^. One study ^64^ provided only BMI classifications and was included solely for food consumption data. Thus, interventions had no significant effect on mean BMI (kg/m^2^) or BMI z-score in the meta-analysis. Although some studies included only in the qualitative synthesis showed reductions, findings were heterogeneous and inconsistent.

95%CI: 95% confidence interval; MD: mean difference; SD: standard deviation. * Boys’ values; ** Girls’ values.

95%CI: 95% confidence interval; MD: mean difference; SD: standard deviation. * Boys’ values; ** Girls’ values.

**Figure 4:**
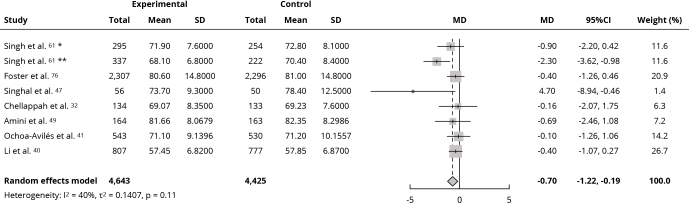
Forest plot of the effect of intervention in food environment school on waist circumference (cm).

Meta-analyses were performed using the WC and body fat data from the selected studies. Mean WC (cm) was significantly reduced by interventions compared to the control group (MD: -0.70; 95%CI: -1.22, -0.19; I^2^ = 40%; low certainty of evidence) (Figure 4). Sensitivity analysis for mean WC showed no change in effect direction after removing studies with high risk of bias, and the result remained significant (Supplementary Material - Figure S5; https://cadernos.ensp.fiocruz.br/static//arquivo/suppl-e00152824_7623.pdf). Among the eight trials ^35^ ^,^ ^46^ ^,^ ^51^ ^,^ ^52^ ^,^ ^54^ ^,^ ^55^ ^,^ ^56^ ^,^ ^80^ included in the qualitative synthesis, four ^35^ ^,^ ^46^ ^,^ ^52^ ^,^ ^54^ found significant WC reductions, including one study ^35^ in which the effect was observed only in boys. Two studies ^55^ ^,^ ^56^ observed increases - one in the control group ^55^ and the other ^56^ in the intervention group - while the remaining studies found no significant changes ^51^ ^,^ ^80^. Overall, despite inconsistencies across individual trials, the meta-analyses suggest that multi-component interventions may be effective in reducing WC in children and adolescents.

95%CI: 95% confidence interval; MD: mean difference; SD: standard deviation. * Boys’ values; ** Girls’ values.

**Figure 5:**
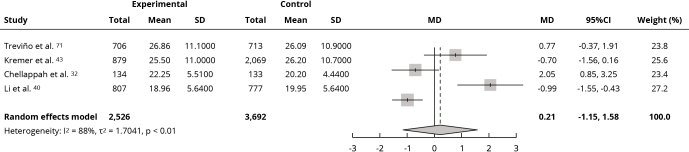
Forest plot of the effect of intervention in food environment school on body fat (%).

Interventions did not affect the mean %BF compared to the control group (MD: 0.21; 95%CI: -1.15, 1.58; I^2^ = 88%; very low certainty of evidence) (Figure 5). Sensitivity analysis did not alter the change in effect direction or significance (Supplementary Material - Figure S6; https://cadernos.ensp.fiocruz.br/static//arquivo/suppl-e00152824_7623.pdf). In the qualitative analysis, seven studies ^46^ ^,^ ^52^ ^,^ ^54^ ^,^ ^70^ ^,^ ^72^ ^,^ ^78^ ^,^ ^80^ reported this outcome, and two ^52^ ^,^ ^78^ found a significant reduction. However, in one study ^78^, this reduction was only observed in boys. Consequently, the intervention does not appear to be effective in reducing %BF in children and adolescents.

95%CI: 95% confidence interval; MD: mean difference; SD: standard deviation.

### Food consumption

Food consumption (fruits, vegetables, unhealthy foods), sugar-sweetened beverages (SSB), and macronutrient or energy intake were assessed as outcomes in 40 studies ^31^ ^,^ ^32^ ^,^ ^33^ ^,^ ^36^ ^,^ ^37^ ^,^ ^39^ ^,^ ^40^ ^,^ ^41^ ^,^ ^42^ ^,^ ^43^ ^,^ ^44^ ^,^ ^45^ ^,^ ^46^ ^,^ ^48^ ^,^ ^49^ ^,^ ^50^ ^,^ ^51^ ^,^ ^53^ ^,^ ^54^ ^,^ ^55^ ^,^ ^56^ ^,^ ^57^ ^,^ ^58^ ^,^ ^59^ ^,^ ^60^ ^,^ ^61^ ^,^ ^62^ ^,^ ^63^ ^,^ ^64^ ^,^ ^66^ ^,^ ^68^ ^,^ ^69^ ^,^ ^70^ ^,^ ^71^ ^,^ ^72^ ^,^ ^73^ ^,^ ^77^ ^,^ ^78^ ^,^ ^80^ ^,^ ^81^ at the end of the interventions. Assessment methods were heterogeneous, using food frequency questionnaires (n = 21) ^31^ ^,^ ^32^ ^,^ ^33^ ^,^ ^36^ ^,^ ^37^ ^,^ ^39^ ^,^ ^40^ ^,^ ^43^ ^,^ ^46^ ^,^ ^48^ ^,^ ^50^ ^,^ ^51^ ^,^ ^53^ ^,^ ^57^ ^,^ ^60^ ^,^ ^61^ ^,^ ^62^ ^,^ ^63^ ^,^ ^64^ ^,^ ^73^ ^,^ ^80^, 24-hour recalls (n = 6) ^41^ ^,^ ^44^ ^,^ ^45^ ^,^ ^49^ ^,^ ^55^ ^,^ ^71^, food diaries (n = 3) ^54^ ^,^ ^56^ ^,^ ^58^, direct observation (n = 2) ^59^ ^,^ ^77^, digital photography (n = 2) ^72^ ^,^ ^78^, and six studies ^42^ ^,^ ^66^ ^,^ ^68^ ^,^ ^69^ ^,^ ^70^ ^,^ ^81^ used more than one measurement method.

Among 15 studies ^32^ ^,^ ^39^ ^,^ ^40^ ^,^ ^41^ ^,^ ^42^ ^,^ ^46^ ^,^ ^48^ ^,^ ^50^ ^,^ ^51^ ^,^ ^56^ ^,^ ^62^ ^,^ ^63^ ^,^ ^64^ ^,^ ^73^ ^,^ ^80^ that evaluated fruit consumption, five reported significant changes. Two studies ^40^ ^,^ ^48^ found a higher fruit intake in the intervention group compared to the control group at follow-up, while one study ^32^ reported a reduction in fruit consumption in the intervention group. In two studies ^41^ ^,^ ^42^, changes were observed only within the intervention group when comparing baseline to follow-up, without statistically significant differences between groups. The remaining studies ^39^ ^,^ ^46^ ^,^ ^50^ ^,^ ^51^ ^,^ ^56^ ^,^ ^62^ ^,^ ^63^ ^,^ ^64^ ^,^ ^73^ ^,^ ^80^ did not report significant differences between groups.

Regarding vegetable consumption, among 14 studies ^32^ ^,^ ^37^ ^,^ ^39^ ^,^ ^40^ ^,^ ^41^ ^,^ ^42^ ^,^ ^48^ ^,^ ^50^ ^,^ ^56^ ^,^ ^62^ ^,^ ^63^ ^,^ ^64^ ^,^ ^73^ ^,^ ^80^, five ^37^ ^,^ ^40^ ^,^ ^48^ ^,^ ^64^ ^,^ ^80^ found higher vegetable intake in the intervention group compared to the control group, while one study ^32^ reported a reduction the intervention group. In two studies ^42^ ^,^ ^56^, the increase was observed only within the intervention group, and in one study ^41^ a decrease was observed within the intervention group, with no statistically significant differences between groups. The remaining studies found no significant differences between groups.

As for unhealthy food consumption, among the nine studies that assessed this outcome ^31^ ^,^ ^41^ ^,^ ^42^ ^,^ ^46^ ^,^ ^50^ ^,^ ^56^ ^,^ ^59^ ^,^ ^64^ ^,^ ^77^, five ^31^ ^,^ ^46^ ^,^ ^50^ ^,^ ^59^ ^,^ ^64^ reported a reduction in the intervention group compared to the control group at follow-up. In three studies ^41^ ^,^ ^56^ ^,^ ^77^, this effect was observed only within the intervention group when comparing baseline to follow-up, rather than between groups. Only one study ^42^ reported an increase in unhealthy food consumption among students with overweight in the intervention group compared to baseline.

Regarding SSB consumption, four studies ^39^ ^,^ ^46^ ^,^ ^50^ ^,^ ^61^ observed a reduction in the intervention group, while six studies ^33^ ^,^ ^43^ ^,^ ^63^ ^,^ ^64^ ^,^ ^80^ ^,^ ^81^ found no differences between groups. However, one study ^56^ reported a higher SSB intake among children in the intervention group at follow-up compared to baseline.

According to five studies ^54^ ^,^ ^55^ ^,^ ^58^ ^,^ ^70^ ^,^ ^72^, school-based interventions reduced total energy intake. However, Majid et al. ^54^ reported similar decreases in energy intake between intervention and control groups compared to baseline, while three studies ^49^ ^,^ ^66^ ^,^ ^68^ found an increase. Four studies ^57^ ^,^ ^73^ ^,^ ^78^ ^,^ ^81^ found no differences between groups. Twelve studies ^41^ ^,^ ^49^ ^,^ ^54^ ^,^ ^55^ ^,^ ^57^ ^,^ ^58^ ^,^ ^66^ ^,^ ^68^ ^,^ ^70^ ^,^ ^72^ ^,^ ^73^ ^,^ ^78^ evaluated energy derived from total fat or total fat in grams per day, five studies ^58^ ^,^ ^66^ ^,^ ^68^ ^,^ ^70^ ^,^ ^72^ observed a reduction in the intervention group, two studies ^49^ ^,^ ^54^ showed increased values throughout the intervention, and five studies ^41^ ^,^ ^47^ ^,^ ^55^ ^,^ ^73^ ^,^ ^78^ observed no differences between groups. Four studies ^55^ ^,^ ^66^ ^,^ ^68^ ^,^ ^72^ also found lower saturated fat intake in schools that implemented changes in the food environment compared to controls or baseline, while two studies ^71^ ^,^ ^78^ did not observe this effect.

Thus, most multi-component interventions did significantly increase fruit consumption or reduce SSB intake. While some studies reported reductions in unhealthy food consumption, total fat, saturated fat, and energy intake, as well as increases in vegetable consumption, others found no significant differences or even reported increases in these outcomes.

### Risk of bias among studies and certainty of evidence

Overall, the risk of bias in cRCTs ranged from low to high. Only seven trials ^33^ ^,^ ^40^ ^,^ ^59^ ^,^ ^68^ ^,^ ^76^ ^,^ ^78^ ^,^ ^81^ were rated as low risk. Most trials ^31^ ^,^ ^32^ ^,^ ^34^ ^,^ ^35^ ^,^ ^36^ ^,^ ^37^ ^,^ ^38^ ^,^ ^39^ ^,^ ^41^ ^,^ ^44^ ^,^ ^45^ ^,^ ^46^ ^,^ ^55^ ^,^ ^57^ ^,^ ^58^ ^,^ ^60^ ^,^ ^61^ ^,^ ^65^ ^,^ ^66^ ^,^ ^67^ ^,^ ^70^ ^,^ ^71^ ^,^ ^80^ presented concerns regarding reported-result selection, deviations from intended interventions, randomization process, and high risk of bias ^42^ ^,^ ^47^ ^,^ ^49^ ^,^ ^63^ ^,^ ^69^ ^,^ ^72^ ^,^ ^73^ ^,^ ^77^ ^,^ ^79^. QE studies were classified as moderate ^48^ ^,^ ^50^ ^,^ ^51^ ^,^ ^52^ ^,^ ^53^ ^,^ ^54^ ^,^ ^62^ ^,^ ^74^ ^,^ ^75^, serious ^43^ ^,^ ^64^, or critical ^56^ risk of bias, with the most frequent domains being outcome measurement, selective outcome reporting, and uncontrolled confounding. Risk of bias assessments are summarized in Figures S7 and S8 (Supplementary Material; https://cadernos.ensp.fiocruz.br/static//arquivo/suppl-e00152824_7623.pdf), while the overall certainty of evidence reported for each outcome is reported in Table S5 (Supplementary Material; https://cadernos.ensp.fiocruz.br/static//arquivo/suppl-e00152824_7623.pdf).

Regarding certainty of evidence, consumption of fruits, vegetables, unhealthy foods, SSB, energy, total fat, and saturated fat was rated as moderate. BMI (z-score) and %BF were rated as very low, while BMI (kg/m^2^) and WC were rated as low.

Publication bias was assessed for the meta-analyses of BMI (kg/m^2^) and BMI z-score. Visual inspection of the funnel plot for the BMI z-score (Supplementary Material - Figure S9a; https://cadernos.ensp.fiocruz.br/static//arquivo/suppl-e00152824_7623.pdf) suggested marked asymmetry, indicating a high potential for publication bias; however, this was not confirmed by Egger’s test (t = 0.50, df = 8, p = 0.6328). The Trim-and-Fill method (Supplementary Material - Figure S9b; https://cadernos.ensp.fiocruz.br/static//arquivo/suppl-e00152824_7623.pdf) imputed missing studies, yielding a corrected MD of -0.043 (95%CI: -0.1347, 0.0487), which reduced the magnitude of the intervention effect while maintaining its direction (p = 0.3581). In contrast, the funnel plot for BMI (kg/m^2^) showed no asymmetry (Supplementary Material - Figure S10; https://cadernos.ensp.fiocruz.br/static//arquivo/suppl-e00152824_7623.pdf), which was consistent with Egger’s test (t = 0.48, df = 15, p = 0.6366). These findings were considered in the GRADE assessment and contributed to downgrading the certainty of evidence for the BMI z-score outcome, which was rated as “strongly suspected” for publication bias.

## Discussion

This systematic review evaluated the impact of school food-environment interventions on adiposity and food consumption outcomes in children and adolescents, including 51 studies. Multi-component interventions that modified the school food environment reduced WC and potentially improved students’ eating habits. However, caution is warranted, since the overall study quality ranged from very low to high. Risk of bias assessments revealed several methodological concerns, such as lack of blinding of outcome assessors, selection bias, and inconsistent reporting, which may have influenced the estimated effects - particularly for anthropometric measures that were often inconsistent or null. Although higher-quality studies tended to demonstrate more robust effects on dietary intake, the observed reduction in WC, despite being statistically significant, was affected by study heterogeneity and potential publication bias.

WC is a recognized indicator of abdominal obesity, specifically reflecting visceral fat accumulation, and is considered an early predictor of cardiometabolic risk in children and adolescents ^82^ ^,^ ^83^ ^,^ ^84^ ^,^ ^85^ ^,^ ^86^ ^,^ ^87^ ^,^ ^88^ ^,^ ^89^. Although moderate correlations exist among BMI, WC, and body fat measures, these anthropometric indicators assess different aspects of body composition and should not be considered equivalent. BMI, despite its widespread use, does not distinguish fat mass from lean mass, nor does it differentiate subcutaneous from visceral fat ^90^. Conversely, WC is a more specific proxy for central adiposity and may be more responsive to early lifestyle modifications, making it a valuable tool for detecting metabolic syndrome in both childhood and adolescence ^83^ ^,^ ^88^. The lack of statistically significant effects observed for BMI and body fat in our meta-analyses may reflect the limited sensitivity of these measures to detect subtle changes over short intervention periods, particularly in samples composed mainly of healthy individuals ^90^ ^,^ ^91^. As highlighted by Grydeland et al. ^91^, more pronounced effects on adiposity-related outcomes may only become evident after longer follow-up periods.

These findings are aligned with previous systematic reviews, which have also reported inconsistent intervention effects on BMI ^22^ ^,^ ^23^ ^,^ ^92^ ^,^ ^93^, while small but favorable changes in WC have been more consistently observed ^90^. Reductions in WC may not only indicate improved adiposity outcomes but also help mitigate the physical, emotional, and economic burdens associated with NCDs. The limited effects observed may partly result from the inclusion of predominantly healthy children in these studies, emphasizing that more substantial benefits may require longer-term interventions and follow-up periods ^90^ ^,^ ^91^.

Regarding food consumption, the results suggest that school-based multi-component interventions positively influenced dietary behaviors by increasing vegetable intake and reducing the consumption of unhealthy foods, total fat, saturated fat, and energy. Previous studies, such as Adom et al. ^92^ and Van Cauwenberghe et al. ^20^, have provided strong evidence of improved fruit and vegetable consumption, while Verstraeten et al. ^17^ reported reductions in fast food intake. Additionally, Gonçalves et al. ^94^ noted that greater availability of healthy foods in schools was associated with a lower risk of obesity, reinforcing the importance of enhancing the school food environment. However, as Micha et al. ^23^ highlight, while multi-component interventions enhance dietary quality, their impact on weight outcomes may not be immediate, emphasizing the need for sustained, long-term strategies.

School-based interventions should adopt a holistic, multifaceted approach that integrates family, school, and community components ^94^ ^,^ ^95^ ^,^ ^96^ ^,^ ^97^ ^,^ ^98^. These interventions should target behavioral modifications by means of lifestyle changes and improvements in the school food environment, such as increasing the availability of healthy foods, restricting unhealthy foods, implementing school policies to regulate food sales, and promoting water consumption ^21^ ^,^ ^22^ ^,^ ^23^ ^,^ ^92^ ^,^ ^93^ ^,^ ^99^. By fostering an environment that supports healthy food choices and regular physical activity, schools can equip young individuals with knowledge and skills needed for a healthier life trajectory ^100^.

Nevertheless, the multi-component approach presents challenges. It is difficult to identify which specific elements drive outcomes, how these components interact, and how to implement multiple actions simultaneously ^97^. Given the complexity and upstream nature of food environment interventions, an inherent dilution bias may occur ^22^. Additionally, factors outside school - such as the home food environment, consumer behavior, attitudes, and personal preferences - can influence results ^22^. A single public policy has limited impact, highlighting the need for comprehensive, long-term strategies to effectively address childhood obesity ^101^.

The duration of interventions is crucial for its success. O’Connor et al. ^102^ suggest that interventions lasting 26 hours or longer can effectively reduce overweight prevalence in children and adolescents. However, establishing a precise recommendation for intervention duration remains challenging due to study heterogeneity and limited comprehensive data.

Several logistical challenges further complicate the assessment and implementation of school food environment interventions. Difficulties with randomization and blinding processes ^103^, barriers related to financial resources, time constraints, school staff ^103^ ^,^ ^104^, and competing school priorities ^105^ can limit implementation. Significant heterogeneity among schools in terms of size, infrastructure, economic and human resources, and sociodemographic characteristics ^106^, along with variations in intervention intensity, duration, frequency, and activity types ^107^ ^,^ ^108^ ^,^ ^109^ ^,^ ^110^ ^,^ ^111^ ^,^ ^112^, has contributed to inconsistent outcomes observed across studies.

The robustness of this systematic review is supported by extensive bibliographic research, protocol registration, no language and date restrictions, rigorous risk-of-bias assessment, evaluation of certainty of evidence, and adherence to PRISMA guidelines throughout screening and data extraction. Most interventions were randomized, which increases the reliability and validity of the findings.

Despite these strengths, some limitations should be acknowledged. The variable quality of the included studies and the heterogeneity in intervention characteristics (intensity, duration, sample size, population) precluded subgroup analyses by students’ age and sex - essential factors given the distinct physiological, cognitive, and socio-emotional developmental stages of children and adolescents. Nevertheless, a key strength is that all interventions were multi-component and specifically targeted the school food environment, which supports their combined analysis. Moreover, the low sensitivity of the search strategy, partly due to the lack of indexed descriptors related to the food environment, may have resulted in the omission of relevant studies.

Additionally, most studies were conducted in high-income countries, and some were excluded from the meta-analysis due to heterogeneity and incomplete data. Finally, most included cRCTs or QE studies did not provide sufficient information to adjust analyses for the intracluster correlation coefficient (ICC). Consequently, pooled estimates may not fully account for within-cluster correlation, potentially underestimating standard errors and overestimating the precision of effect sizes. To enhance the reliability of future evidence syntheses, primary studies should report ICCs and provide cluster-adjusted effect estimates.

## Conclusion

Interventions targeting the school food environment offer limited evidence on their effects on adiposity and food consumption in children and adolescents. Multi-component interventions have reduced WC and may have improved dietary behaviors, although BMI results were inconsistent. The wide variability in study quality, intervention design, and implementation prevents definitive conclusions on their long-term effectiveness. Methodological limitations - such as heterogeneity in intervention components, duration, and adherence - may obscure their true impact. Improving study designs, standardizing approaches, and securing policy support are crucial to better assess and enhance these strategies for lasting health improvements.

Moreover, multi-component interventions must actively engage the entire school community to promote healthy food choices, nutrition education, and a wellness-focused lifestyle. Integrating nutrition education into the school curriculum and implementing regulatory policies that restrict unhealthy food sales and advertising while promoting healthier alternatives are fundamental for sustained impact.

Effective, policy-supported interventions foster healthier school environments and establish the foundation for lifelong healthy habits. A coordinated effort among schools, communities, policymakers, and public health stakeholders is essential for meaningful change and a healthier future for younger generations.

## Data Availability

The sources of information used in the study are indicated in the body of the article.
