# Supplementary material for: Impact of multi-component school food environment interventions on adiposity and food consumption in children and adolescents: systematic review and meta-analysis
Source: Cad Saude Publica. 2025 Dec 1;41(11):e00152824. doi: 10.1590/0102-311XEN152824 (PMC12688213; doi:10.1590/0102-311XEN152824)
Supplement: Supplementary Material [file 1678-4464-csp-41-11-EN152824-s.pdf]

SUPPLEMENTARY MATERIAL

Table S1 Preferred Reporting Items for Systematic Reviews and Meta-Analyses (PRISMA) 2020 Checklist.[1]

Section and Topic	Item #	Checklist item	Location where item is reported
**TITLE**			
Title	1	Identify the report as a systematic review.	Page 1, Lines 1-3
**ABSTRACT**			
Abstract	2	See the PRISMA 2020 for Abstracts checklist.	Title page
**INTRODUCTION**			
Rationale	3	Describe the rationale for the review in the context of existing knowledge.	Page 1, Lines 18-28
Objectives	4	Provide an explicit statement of the objective(s) or question(s) the review addresses.	Page 1, Lines 28-30
**METHODS**			
Eligibility criteria	5	Specify the inclusion and exclusion criteria for the review and how studies were grouped for the syntheses.	Page 2, Lines 52-64 (Table S3)
Information sources	6	Specify all databases, registers, websites, organisations, reference lists and other sources searched or consulted to identify studies. Specify the date when each source was last searched or consulted.	Page 2, Lines 43-46
Search strategy	7	Present the full search strategies for all databases, registers and websites, including any filters and limits used.	Page 2, Lines 46-49 (Table S2)
Selection process	8	Specify the methods used to decide whether a study met the inclusion criteria of the review, including how many reviewers screened each record and each report retrieved, whether they worked independently, and if applicable, details of automation tools used in the process.	Pages 3, Lines 67-71
Data collection process	9	Specify the methods used to collect data from reports, including how many reviewers collected data from each report, whether they worked independently, any processes for obtaining or confirming data from study investigators, and if applicable, details of automation tools used in the process.	Page 3, Lines 71-78
Data items	10a	List and define all outcomes for which data were sought. Specify whether all results that were compatible with each outcome domain in each study were sought (e.g. for all measures, time points, analyses), and if not, the methods used to decide which results to collect.	Page 2, Lines 56-57
	10b	List and define all other variables for which data were sought (e.g. participant and intervention characteristics, funding sources). Describe any assumptions made about any missing or unclear information.	Page 2, Lines 52-59
Study risk of bias assessment	11	Specify the methods used to assess risk of bias in the included studies, including details of the tool(s) used, how many reviewers assessed each study and whether they worked independently, and if applicable, details of automation tools used in the process.	Page 4, Lines 105-125
Effect measures	12	Specify for each outcome the effect measure(s) (e.g. risk ratio, mean difference) used in the synthesis or presentation of results.	Page 3, Lines 85-90
Synthesis methods	13a	Describe the processes used to decide which studies were eligible for each synthesis (e.g. tabulating the study intervention characteristics and comparing against the planned groups for each synthesis (item #5)).	Page 3, Lines 67-78
	13b	Describe any methods required to prepare the data for presentation or synthesis, such as handling of missing summary statistics, or data conversions.	Page 3, Lines 86-90
	13c	Describe any methods used to tabulate or visually display results of individual studies and syntheses.	Page 3, Lines 73-78
	13d	Describe any methods used to synthesize results and provide a rationale for the choice(s). If meta-analysis was performed, describe the model(s), method(s) to identify the presence and extent of statistical heterogeneity, and software package(s)	Page 3, Lines 81-84; 91-97

		used.	
	13e	Describe any methods used to explore possible causes of heterogeneity among study results (e.g. subgroup analysis, meta-regression).	Pages 3-4, Lines 92-102
	13f	Describe any sensitivity analyses conducted to assess robustness of the synthesized results.	Page 3, Lines 97-99
Reporting bias assessment	14	Describe any methods used to assess risk of bias due to missing results in a synthesis (arising from reporting biases).	Page 4, Lines 104-122
Certainty assessment	15	Describe any methods used to assess certainty (or confidence) in the body of evidence for an outcome.	Page 4, Lines 123-125
**RESULTS**			
Study selection	16a	Describe the results of the search and selection process, from the number of records identified in the search to the number of studies included in the review, ideally using a flow diagram.	Pages 4-5, Lines 128-134 (Figure 1)
	16b	Cite studies that might appear to meet the inclusion criteria, but which were excluded, and explain why they were excluded.	Page 5, Lines 133-134 (Table S4)
Study characteristics	17	Cite each included study and present its characteristics.	Page 5, Lines 137-166 (Table 1)
Risk of bias in studies	18	Present assessments of risk of bias for each included study.	Pages 8-9, Lines 261-269 (Figure S7 and S8)
Results of individual studies	19	For all outcomes, present, for each study: (a) summary statistics for each group (where appropriate) and (b) an effect estimate and its precision (e.g. confidence/credible interval), ideally using structured tables or plots.	Pages 6-8, Lines 169-258 (Figure S1, Table 1)
Results of syntheses	20a	For each synthesis, briefly summarise the characteristics and risk of bias among contributing studies.	Pages 6-8, Lines 169-258
	20b	Present results of all statistical syntheses conducted. If meta-analysis was done, present for each the summary estimate and its precision (e.g. confidence/credible interval) and measures of statistical heterogeneity. If comparing groups, describe the direction of the effect.	Pages 6-8, Lines 169-258 (Figures 2, 3, 4, S2)
	20c	Present results of all investigations of possible causes of heterogeneity among study results.	Pages 6-8, Lines 169-258
	20d	Present results of all sensitivity analyses conducted to assess the robustness of the synthesized results.	Pages 6-8, Lines 169-258 (Figures S3, S4, S5, S6)
Reporting biases	21	Present assessments of risk of bias due to missing results (arising from reporting biases) for each synthesis assessed.	Pages 9, Lines 261-269/274-284 (Figures S9 e S10)
Certainty of evidence	22	Present assessments of certainty (or confidence) in the body of evidence for each outcome assessed.	Page 8, Lines 267-273 (Table S5)
**DISCUSSION**			
Discussion	23a	Provide a general interpretation of the results in the context of other evidence.	Pages 9-12, Lines 287-351; 385-393
	23b	Discuss any limitations of the evidence included in the review.	Pages 9-11, Lines 290-297; 339-346
	23c	Discuss any limitations of the review processes used.	Pages 11-12, Lines 365-382
	23d	Discuss implications of the results for practice, policy, and future research.	Page 12, Lines 394-402
**OTHER INFORMATION**			
Registration and protocol	24a	Provide registration information for the review, including register name and registration number, or state that the review was not registered.	Page 2, Lines 39-40
	24b	Indicate where the review protocol can be accessed, or state that a protocol was not prepared.	Page 2, Lines 39-40
	24c	Describe and explain any amendments to information provided at registration or in the protocol.	Page 2, Lines 39-40
Support	25	Describe sources of financial or non-financial support for the review, and the role of the funders or sponsors in the review.	not applicable (not in main document)
Competing interests	26	Declare any competing interests of review authors.	not applicable (not in main document)
Availability of data, code and other materials	27	Report which of the following are publicly available and where they can be found: template data collection forms; data extracted from included studies; data used for all analyses; analytic code; any other materials used in the review.	Supplemental Material

Table S2 Search strategy for systematic review.

CENTRAL
"Child" or "Children" or "Child, Preschool" or "Preschool Child" or "Preschool Children" or "Adolescent" or "Adolescents" or "Adolescence" or "Teens" or "Teen" or "Teenagers" or "Teenager" or "Youth" or "Youths" or "Students" or "Student" or "Schools" or "School" or "Primary Schools" or "Primary School" or "School, Primary" or "Schools, Primary" or "Schools, Secondary" or "School, Secondary" or "Secondary School" or "Secondary Schools" in Title Abstract Keyword AND "Weight Reduction Programs" OR "Reduction Programs, Weight" OR "Weight Reduction Program" OR "Weight Loss Programs" OR "Weight Loss Program" OR "Food environment" OR "Environment Intervention" OR "Environment Interventions" OR "Food Environment Intervention" OR "Food Environment Interventions" OR "Nutrition Intervention" OR "Nutrition Interventions" OR "Health Intervention" OR "Health Interventions" OR "Store Intervention" OR "Nutrition Policy" OR "Nutrition Policies" OR "Food Policy" OR "Food Policies" OR "Government Regulation" OR "legislation" OR "legislations" OR "law" OR "laws" OR "ban" OR "bans" OR "nutrition policy" OR "school food policy" OR "cafeteria-based intervention" OR "health promotion" OR "school-based program" OR "food service intervention" OR "foodservice intervention" OR "foodservice policy" OR "food service policy" OR "foodservice program" OR "foodservice programs" OR "Health Policy" OR "Health Behavior" OR "Environmental Policy" OR "Nutrition Policy" OR "Health Promotion" OR "Food Industry" OR "Food Industries" OR "Industries, Food" OR "Industry, Food" OR "Dietary Fats" OR "Fats, Dietary" OR "Dietary Fat" OR "Fat, Dietary" OR "nudging" OR "lunch program" OR "nutrition program" in Title Abstract Keyword AND "School Canteens" OR "School Canteen" OR "Food Sales" OR "Food Sale" OR "Store School" OR "Cafeterias" OR "Cafeteria" OR "School Neighborhood" OR "Snack Bar" OR "Snack Bars" OR "Snack, Bars" OR "Tuck Shops" OR "Tuck Shop" OR "Tuck, Shop" OR "Kiosks" OR "Kiosk" OR "Canteens" OR "Canteen" OR "Food Vendors" OR "Food Vendor" OR "Vending Machines" OR "Vending Machine" OR "Informal Labor Market" OR "Informal Labor Markets" OR "Street Seller" OR "Street Sellers" in Title Abstract Keyword
**CLINICAL TRIALS**
Condition or disease: child OR children OR students OR schools OR child, preschool OR adolescent OR teens OR youth OR students OR schools OR primary schools OR secondary schools<br><br>Other terms: canteen OR cafeteria OR vending machine OR snack bar OR store school OR tuck shop OR kiosks<br><br>Intervention/treatment: weight Loss program OR nutrition policy OR school-based intervention OR health promotion OR health care policy OR government regulation OR health behavior OR school health service OR food industry OR nudging OR nutrition program OR food environment
**EMBASE**
#5 = #1 AND #4<br>318<br>#4 = #2 AND #3<br>464<br>#3<br>1,321,317<br>'weight loss program'/syn OR 'nutrition policy'/syn OR 'government regulation'/syn OR 'school based intervention'/syn OR 'health promotion'/syn OR 'health care policy'/syn OR 'environmental policy'/syn OR 'health behavior'/syn OR 'school health service/syn' OR 'food industry/syn' OR 'nudging' OR 'nutrition programs' OR 'food environment' OR 'government regulation/syn' OR 'food legislation/syn' OR 'law'<br>#2<br>2,494<br>'canteen'/syn OR 'cafeteria diet'/syn OR 'vending machine'/syn<br>#1<br>6,176,506<br>'adolescent'/syn OR 'child'/syn OR 'juvenile'/syn OR 'student'/syn OR 'primary schools'/syn OR 'high school'/syn OR 'public school'/syn OR 'private school'/syn OR 'school child/syn' OR 'kindergarten/syn' OR 'middle school/syn' OR 'middle school student/syn' OR 'high school student/syn' OR 'elementary student/syn' OR 'preschool child/syn' OR 'preschool'
**MEDLINE (via PubMed)**
"Child"[MeSH] OR "Children"[Title/Abstract] OR "Child, Preschool"[MeSH] OR "Preschool Child"[Title/Abstract] OR "Children, Preschool"[Title/Abstract] OR "Preschool Children"[Title/Abstract] OR "Adolescent"[MeSH] OR "Adolescents"[Title/Abstract] OR "Adolescence"[Title/Abstract] OR "Teens"[Title/Abstract] OR "Teen"[Title/Abstract] OR "Teenagers"[Title/Abstract] OR "Teenager"[Title/Abstract] OR "Youth"[Title/Abstract] OR "Youths"[Title/Abstract] OR "Adolescents, Female"[Title/Abstract] OR "Adolescent, Female"[Title/Abstract] OR "Female Adolescent"[Title/Abstract] OR "Female Adolescents"[Title/Abstract] OR "Adolescents, Male"[Title/Abstract] OR "Adolescent, Male"[Title/Abstract] OR "Male Adolescent"[Title/Abstract] OR "Male Adolescents"[Title/Abstract] OR "Students"[MeSH] OR "Schools"[MeSH] OR "School"[Title/Abstract] OR "Primary Schools"[Title/Abstract] OR "Primary School"[Title/Abstract] OR "School, Primary"[Title/Abstract] OR "Schools, Primary"[Title/Abstract] OR "Schools, Secondary"[Title/Abstract] OR "School, Secondary"[Title/Abstract] OR "Secondary School"[Title/Abstract] OR "Secondary Schools"[Title/Abstract]<br>AND<br>"Weight Reduction Programs"[MeSH] OR "Program, Weight Reduction"[Title/Abstract] OR "Programs, Weight Reduction"[Title/Abstract] OR "Reduction Program, Weight"[Title/Abstract] OR "Weight Reduction Program"[Title/Abstract] OR "Weight Loss Programs"[Title/Abstract] OR "Loss Program, Weight"[Title/Abstract] OR "Loss Programs, Weight"[Title/Abstract]

OR "Program, Weight Loss"[Title/Abstract] OR "Programs, Weight Loss"[Title/Abstract] OR "Weight Loss Program"[Title/Abstract] OR "Environment Intervention"[Title/Abstract] OR "Environment, Intervention"[Title/Abstract] OR "Food environment"[Title/Abstract] OR "Environment Interventions"[Title/Abstract] OR "Environment, Interventions"[Title/Abstract] OR "Food Environment Intervention"[Title/Abstract] OR "Food, Environment Intervention"[Title/Abstract] OR "Food Environment Interventions"[Title/Abstract] OR "Food, Environment Interventions"[Title/Abstract] OR "Nutrition Intervention"[Title/Abstract] OR "Nutrition, Intervention"[Title/Abstract] OR "Nutrition Interventions"[Title/Abstract] OR "Nutrition, Interventions"[Title/Abstract] OR "Health Intervention"[Title/Abstract] OR "Health, Intervention"[Title/Abstract] OR "Health Interventions"[Title/Abstract] OR "Health, Interventions"[Title/Abstract] OR "Store Intervention"[Title/Abstract] OR "Store, Intervention"[Title/Abstract] OR ("Food Retail"[Title/Abstract] AND "Intervention"[Title/Abstract]) OR ("Food, Retail"[Title/Abstract] AND "Intervention"[Title/Abstract]) OR ("Food Retail"[Title/Abstract] AND "Interventions"[Title/Abstract]) OR ("Food, Retail"[Title/Abstract] AND "Interventions"[Title/Abstract]) OR "Nutrition Policy"[MeSH] OR "Nutrition Policies"[Title/Abstract] OR "Policies, Nutrition"[Title/Abstract] OR "Policy, Nutrition"[Title/Abstract] OR "Food Policy"[Title/Abstract] OR "Food Policies"[Title/Abstract] OR "Policies, Food"[Title/Abstract] OR "Policy, Food"[Title/Abstract] OR "Government Regulation"[MeSH] OR "Legislation, Food"[MeSH] OR "legislation"[Title/Abstract] OR "legislations"[Title/Abstract] OR "law"[Title/Abstract] OR "laws"[Title/Abstract] OR "ban"[Title/Abstract] OR "bans"[Title/Abstract] OR "school food policy"[Title/Abstract] OR ("promoting"[Title/Abstract] AND "school environment"[Title/Abstract]) OR "cafeteria-based intervention"[Title/Abstract] OR "health promotion"[Title/Abstract] OR "school-based program"[Title/Abstract] OR "food service intervention"[Title/Abstract] OR "foodservice program"[Title/Abstract] OR "foodservice programs"[Title/Abstract] OR "Health Policy"[MeSH] OR "Health Behavior"[MeSH] OR "Environmental Policy"[MeSH] OR "School Health Services"[MeSH] OR "Health Promotion"[MeSH] OR "Food Industry"[MeSH] OR "Food Industries"[Title/Abstract] OR "Industries, Food"[Title/Abstract] OR "Industry, Food"[Title/Abstract] OR "Dietary Fats"[MeSH] OR "Fats, Dietary"[Title/Abstract] OR "Dietary Fat"[Title/Abstract] OR "Fat, Dietary"[Title/Abstract] OR "nudging"[Title/Abstract] OR "lunch program"[Title/Abstract] OR "nutrition program"[Title/Abstract]

AND

"School Canteens"[Title/Abstract] OR "School Canteen"[Title/Abstract] OR "School, Canteens"[Title/Abstract] OR "School, Canteen"[Title/Abstract] OR "Food Sales"[Title/Abstract] OR "Food Sale"[Title/Abstract] OR "Food, Sales"[Title/Abstract] OR "Food, Sale"[Title/Abstract] OR "Cafeterias"[Title/Abstract] OR "Cafeteria"[Title/Abstract] OR "School Neighborhood"[Title/Abstract] OR "School, Neighborhood"[Title/Abstract] OR "Snack Bar"[Title/Abstract] OR "Snack Bars"[Title/Abstract] OR "Snack, Bar"[Title/Abstract] OR "Snack, Bars"[Title/Abstract] OR "Tuck Shops"[Title/Abstract] OR "Tuck Shop"[Title/Abstract] OR "Tuck, Shops"[Title/Abstract] OR "Tuck, Shop"[Title/Abstract] OR "Kiosks"[Title/Abstract] OR "Kiosk"[Title/Abstract] OR "Canteens"[Title/Abstract] OR "Canteen"[Title/Abstract] OR "Food Vendors"[Title/Abstract] OR "Food Vendor"[Title/Abstract] OR "Food, Vendors"[Title/Abstract] OR "Food, Vendor"[Title/Abstract] OR "Vending Machines"[Title/Abstract] OR "Vending Machine"[Title/Abstract] OR "Vending, Machines"[Title/Abstract] OR "Vending, Machine"[Title/Abstract] OR "Informal Labor Market"[Title/Abstract] OR "Informal, Labor Market"[Title/Abstract] OR "Street Sellers"[Title/Abstract] OR "Street, Sellers"[Title/Abstract]

SciELO

("Child" or "Children" or "Child, Preschool" or "Preschool Child" or "Preschool Children" or "Children, Preschool" or "Adolescent" or "Adolescents" or "Adolescence" or "Teens" or "Teen" or "Teenagers" or "Teenager" or "Youth" or "Youths" or "Students" or "Student" or "Schools" or "School" or "Primary Schools" or "Primary School" or "School, Primary" or "Schools, Primary" or "Schools, Secondary" or "School, Secondary" or "Secondary School" or "Secondary Schools") AND ("Weight Reduction Programs" OR "Reduction Programs, Weight" OR "Weight Reduction Program" OR "Weight Loss Programs" OR "Weight Loss Program" OR “Food environment” OR "Environment Intervention" OR "Environment Interventions" OR "Food Environment Intervention" OR "Food Environment Interventions" OR "Nutrition Intervention" OR "Nutrition Interventions" OR "Health Intervention" OR "Health Interventions" OR "Store Intervention" OR "Nutrition Policy" OR "Nutrition Policies" OR "Food Policy" OR "Food Policies" OR "Government Regulation" OR "legislation" OR "legislations" OR "law" OR "laws" OR "ban" OR "bans" OR "nutrition policy" OR "school food policy" OR "cafeteria-based intervention" OR "health promotion" OR "school-based program" OR "food service intervention" OR "foodservice intervention" OR "foodservice policy" OR "food service policy" OR "foodservice program" OR "foodservice programs" OR "Health Policy" OR "Health Behavior" OR "Environmental Policy" OR "Nutrition Policy" OR "Health Promotion" OR "Food Industry" OR "Food Industries" OR "Industries, Food" OR "Industry, Food" OR "Dietary Fats" OR "Fats, Dietary" OR "Dietary Fat" OR "Fat, Dietary" OR "nudging" OR "lunch program" OR "nutrition program") AND ("School Canteens" OR "School Canteen" OR "Food Sales" OR "Food Sale" OR "Store School" OR "Cafeterias" OR "Cafeteria" OR "School Neighborhood" OR "Snack Bar" OR "Snack Bars" OR "Snack, Bars" OR "Tuck Shops" OR "Tuck Shop" OR "Tuck, Shop" OR "Kiosks" OR "Kiosk" OR "Canteens" OR "Canteen" OR "Food Vendors" OR "Food Vendor" OR "Vending Machines" OR "Vending Machine" OR "Informal Labor Market" OR "Informal Labor Markets" OR "Street Seller" OR "Street Sellers")

Scopus

(TITLE-ABS-KEY ("Child" OR "Children" OR "Child, Preschool" OR "Preschool Child" OR "Preschool Children" OR "Children, Preschool" OR "Adolescent" OR "Adolescents" OR "Adolescence" OR "Teens" OR "Teen" OR "Teenagers" OR "Teenager" OR "Youth" OR "Youths" OR "Students" OR "Student" OR "Schools" OR "School" OR "Primary Schools" OR "Primary School" OR "School, Primary" OR "Schools, Primary" OR "Schools, Secondary" OR "School, Secondary" OR "Secondary School" OR "Secondary Schools") AND TITLE-ABS-KEY ("Weight Reduction Programs" OR "Reduction Programs, Weight" OR "Weight Reduction Program" OR "Weight Loss Programs" OR "Weight Loss Program" OR "Food environment" OR "Environment Intervention" OR "Environment Interventions" OR "Food Environment Intervention" OR "Food Environment Interventions" OR "Nutrition Intervention" OR "Nutrition Interventions" OR "Health Intervention" OR "Health Interventions" OR "Store Intervention" OR "Nutrition Policy" OR "Nutrition Policies" OR "Food Policy" OR "Food Policies" OR "Government Regulation" OR "legislation" OR "legislations" OR "law" OR "laws" OR "ban" OR "bans" OR "nutrition policy" OR "school food

policy" OR "cafeteria-based intervention" OR "health promotion" OR "school-based program" OR "food service intervention" OR "foodservice intervention" OR "foodservice policy" OR "food service policy" OR "foodservice program" OR "foodservice programs" OR "Health Policy" OR "Health Behavior" OR "Environmental Policy" OR "Nutrition Policy" OR "Health Promotion" OR "Food Industry" OR "Food Industries" OR "Industries, Food" OR "Industry, Food" OR "Dietary Fats" OR "Fats, Dietary" OR "Dietary Fat" OR "Fat, Dietary" OR "nudging" OR "lunch program" OR "nutrition program") AND TITLE-ABS-KEY ("School Canteens" OR "School Canteen" OR "Food Sales" OR "Food Sale" OR "Store School" OR "Cafeterias" OR "Cafeteria" OR "School Neighborhood" OR "Snack Bar" OR "Snack Bars" OR "Snack, Bars" OR "Tuck Shops" OR "Tuck Shop" OR "Tuck, Shop" OR "Kiosks" OR "Kiosk" OR "Canteens" OR "Canteen" OR "Food Vendors" OR "Food Vendor" OR "Vending Machines" OR "Vending Machine" OR "Informal Labor Market" OR "Informal Labor Markets" OR "Street Seller" OR "Street Sellers"))

Web of Science

#3
AND
#2
AND
#1

3

TS=("Child" OR "Children" OR "Child, Preschool" OR "Preschool Child" OR "Children, Preschool" OR "Preschool Children" OR "Adolescent" OR "Adolescents" OR "Adolescence" OR "Teens" OR "Teen" OR "Teenagers" OR "Teenager" OR "Youth" OR "Youths" OR "Adolescents, Female" OR "Adolescent, Female" OR "Female Adolescent" OR "Female Adolescents" OR "Adolescents, Male" OR "Adolescent, Male" OR "Male Adolescent" OR "Male Adolescents" OR "Students" OR "Schools" OR "School" OR "Primary Schools" OR "Primary School" OR "School, Primary" OR "Schools, Primary" OR "Schools, Secondary" OR "School, Secondary" OR "Secondary School" OR "Secondary Schools")

2

TS=("School Canteens" OR "School Canteen" OR "School, Canteens" OR "School, Canteen" OR "Food Sales" OR "Food Sale" OR "Food, Sales" OR "Food, Sale" OR "Cafeterias" OR "Cafeteria" OR "School Neighborhood" OR "School, Neighborhood" OR "Snack Bar" OR "Snack Bars" OR "Snack, Bar" OR "Snack, Bars" OR "Tuck Shops" OR "Tuck Shop" OR "Tuck, Shops" OR "Tuck, Shop" OR "Kiosks" OR "Kiosk" OR "Canteens" OR "Canteen" OR "Food Vendors" OR "Food Vendor" OR "Food, Vendors" OR "Food, Vendor" OR "Vending Machines" OR "Vending Machine" OR "Vending, Machines" OR "Vending, Machine" OR "Informal Labor Market" OR "Informal, Labor Markets" OR "Informal, Labor Market" OR "Street Sellers" OR "Street, Sellers")

1

TS=("Weight Reduction Programs" OR "Program, Weight Reduction" OR "Programs, Weight Reduction" OR "Reduction Program, Weight" OR "Reduction Programs, Weight" OR "Weight Reduction Program" OR "Weight Loss Programs" OR "Loss Program, Weight" OR "Loss Programs, Weight" OR "Program, Weight Loss" OR "Programs, Weight Loss" OR "Weight Loss Program" OR “Food environment” OR "Environment Intervention" OR "Environment, Intervention" OR "Environment Interventions" OR "Environment, Interventions" OR "Food Environment Intervention" OR "Food, Environment Intervention" OR "Food Environment Interventions" OR "Food, Environment Interventions" OR "Nutrition Intervention" OR "Nutrition, Intervention" OR "Nutrition Interventions" OR "Nutrition, Interventions" OR "Health Intervention" OR "Health, Intervention" OR "Health Interventions" OR "Health, Interventions" OR "Store Intervention" OR "Store, Intervention" OR ("Food Retail" AND "Intervention") OR ("Food, Retail" AND "Intervention") OR ("Food Retail" AND "Interventions") OR ("Food, Retail" AND "Interventions") OR "Nutrition Policy" OR "Nutrition Policies" OR "Policies, Nutrition" OR "Policy, Nutrition" OR "Food Policy" OR "Food Policies" OR "Policies, Food" OR "Policy, Food" OR "Government Regulation" OR "Legislation, Food" OR "legislation" OR "legislations" OR "law" OR "laws" OR "ban" OR "bans" OR "school food policy" OR ("promoting" AND "school environment") OR "cafeteria-based intervention" OR "health promotion" OR "school-based program" OR "food service intervention" OR "foodservice program" OR "foodservice programs" OR "Health Policy" OR "Health Behavior" OR "Environmental Policy" OR "Health Service, School" OR "School Health Service" OR "Service, School Health" OR "School-Based Services" OR "School Based Services" OR "School-Based Service" OR "Services, School-Based" OR "Services, School Health" OR "School-Based Health Services" OR "Health Service, School-Based" OR "Health Services, School-Based" OR "School Based Health Services" OR "School-Based Health Service" OR "Services, School-Based Health" OR "Health Services, School" OR "School Health Promotion" OR "Health Promotion, School" OR "Promotion, School Health" OR "Health Promotion" OR "Promotion, Health" OR "Promotions, Health" OR "Promotion of Health" OR "Health Promotions" OR "Promotional Items" OR "Item, Promotional" OR "Promotional Item" OR "Wellness Programs" OR "Program, Wellness" OR "Programs, Wellness" OR "Wellness Program" OR "Health Campaigns" OR "Campaign, Health" OR "Campaigns, Health" OR "Health Campaign" OR "Food Services" OR "Services, Food" OR "Food Service" OR "Service, Food" OR "Meals on Wheels" OR "Food Standards" OR "Food Industry" OR "Food Industries" OR "Industries, Food" OR "Industry, Food" OR "Dietary Fats" OR "Fats, Dietary" OR "Dietary Fat" OR "Fat, Dietary" OR "nudging" OR "lunch program" OR "nutrition program")

Table S3 Population, Intervention/Exposure, Comparators, Outcome, and Study (PICOS) criteria for inclusion and exclusion of studies from November 2023 literature search.

Category	Inclusion criteria	Exclusion criteria
**Participants**	Students including children (> 2 years old) and adolescents (<19 years old)	Children (< 2 years old), adults (>19 years old) and elderly people
**Intervention/ exposure**	School food environment (internal environment and surroundings) - economic, nutritional, school ambiance, legislation and regulations for the sale of food in schools	Interventions only educational and not in the food environment
**Comparator**	Non-exposed to the intervention control group	None
**Outcomes**	Adiposity (body mass index, body fat percentage, waist circumference) and changes in food consumption	Studies only presenting food consumption outcomes
**Study design**	Cluster randomized controlled trials, quasi-experimental studies, and field trials.<br>No period or language restrictions were used	Observational studies (cohort studies, cross-sectional studies, case-control studies), prospective studies, systematic reviews and meta-analyses, letters, editorials, and articles repeating information from a previously included population

Table S4 Exclusion of the 43 complete texts from November 2023 search.

Author	Year	Title	Reasons for exclusions
Almughamisi et al.[2]	2020	Promoting healthy school food environments in Jeddah, Saudi Arabia: An adolescent led canteen intervention appeals care providers	Wrong study design
Andrade et al.[3]	2014	A school-based intervention improves physical fitness in Ecuadorian adolescents: a cluster-randomized controlled trial	Not food environment intervention
Barbosa Filho et al.[4]	2019	Effect of a multicomponent intervention on lifestyle factors among Brazilian adolescents from low human development index areas: a cluster-randomized controlled trial	Outcomes not assessed
Berenson et al.[5]	2010	Cardiovascular health promotion for children: a model for a Parish (County)-wide program (Implementation and Preliminary Results)	Wrong study design
Birnbaum et al.[6]	2002	Are differences in exposure to a multicomponent school-based intervention associated with varying dietary outcomes in adolescents?	Outcomes not assessed
Coleman et al.[7]	2011	Changing nutrition policies and environments in low-income schools using implementation models: the healthy options for nutrition environments in schools (ONES) intervention	Outcomes not assessed
Damsgaard et al.[8]	2012	Design of the OPUS School Meal Study: a randomised controlled trial assessing the impact of serving school meals based on the New Nordic Diet	Wrong study design
Davee et al.[9]	2005	The vending and à la carte policy intervention in maine public high schools	Outcomes not assessed
Florence et al.[10]	2020	Physical and nutrition education intervention improves body weight status of adolescents in Uasin Gishu county, Kenya: A cluster-randomized controlled trial	Not food environment intervention
Foster et al.[11]	2007	Middle-School Based Primary Prevention Trial of Type 2 Diabetes	Appropriate data unavailable
French et al.[12]	1997	Pricing strategy to promote fruit and vegetable purchase in high school cafeterias	Outcomes not assessed
French et al.[13]	2004	An environmental intervention to promote lower-fat food choices in secondary schools: outcomes of the TACOS Study	Outcomes not assessed
Fu et al.[14]	2019	School accreditation scheme reduces childhood obesity in Hong Kong	Wrong study design
Gallotta et al.[15]	2016	Effects of combined physical education and nutritional programs on schoolchildren's healthy habits	Not food environment intervention
Haerens et al.[16]	2006	Evaluation of a 2-year physical activity and healthy eating intervention in middle school children	Outcomes not assessed
Lane et al.[17]	2018	Wellness Champions for Change," a multi-level intervention to improve school-level implementation of local wellness policies: Study protocol for a cluster randomized trial	Appropriate data unavailable
Hoppu et al.[18]	2010	The diet of adolescents can be improved by school intervention	Outcomes not assessed
Hussain et al.[19]	2020	Three years follow-up (cohort) study via population-based intervention on adolescent and childhood obesity and overweight at schools setting	Appropriate data unavailable
Leme et al.[20]	2018	Sustained impact of the "Healthy Habits, Healthy Girls – Brazil" school-based randomized controlled trial for adolescents living in low-income communities	Not food environment intervention
Li et al.[21]	2014	Effectiveness of a school-based physical activity intervention on obesity in school children: a nonrandomized controlled trial	Not food environment intervention
Lytle[22]	1998	Lessons from the Child and Adolescent Trial for Cardiovascular Health (CATCH): interventions with children	Wrong study design
Lytle et al.[23]	2004	School-based approaches to affect adolescents' diets: results from the TEENS study	Outcomes not assessed
Majid et al.[24]	2018	A school-based study to improve healthy eating habits amongst Malaysian teenagers	Appropriate data unavailable
Moreno et al.[25]	2021	A cluster-randomized controlled trial of an elementary school drinking water access and promotion intervention: rationale, study design, and protocol	Appropriate data unavailable

Nik Rosmawati et al.[26]	2018	Evaluating the implementation of a canteen-based food nutrition intervention among schoolchildren: a prospective intervention study	Not food environment intervention
Ochoa-Alviles et al.[27]	2017	Effect of the school-based health promotion intervention activital on dietary intake and waist circumference: A cluster randomized controlled trial	Appropriate data unavailable
Olsen et al.[28]	2021	Primary prevention of fat and weight gain among obesity susceptible healthy weight preschool children. Main results from the "Healthy Start" randomized controlled intervention	Not food environment intervention
Perry et al.[29]	2004	A randomized school trial of environmental strategies to encourage fruit and vegetable consumption among children	Outcomes not assessed
Pramesthi et al.[30]	2019	Intervention of the nutrition goes to school program for adolescents in Malang district, East Java: Baseline report	Appropriate data unavailable
Ramos[31]	2019	Education for health. Nutrition and gastronomy in the Autonomous Cities of Melilla and Ceuta	Wrong study design
Rerksuppaphol et al.[32]	2017	Internet based obesity prevention program for Thai school children - a randomized control trial	Not food environment intervention
Schwartz et al.[33]	2016	Effect of a School-Based Water Intervention on Child Body Mass Index and Obesity	Wrong study design
Serra-Paya et al.[34]	2015	Effectiveness of a multi-component intervention for overweight and obese children (Nereu Program): a randomized controlled trial	Not food environment intervention
Sichieri et al.[35]	2009	School randomised trial on prevention of excessive weight gain by discouraging students from drinking sodas	Not food environment intervention
Singh et al.[36]	2006	Design of the Dutch Obesity Intervention in Teenagers (NRG-DOiT): systematic development, implementation and evaluation of a school-based intervention aimed at the prevention of excessive weight gain in adolescents	Appropriate data unavailable
Schuh[37]	2015	Program for health promotion in schools of public elementary school in the state of Rio Grande do Sul	Appropriate data unavailable
Teo et al.[38]	2019	School-based intervention that integrates nutrition education and supportive healthy school food environment among Malaysian primary school children: a study protocol	Appropriate data unavailable
Turnin et al.[39]	2015	Effect of Nutritional Intervention on Food Choices of French Students in Middle School Cafeterias, Using an Interactive Educational Software Program (Nutri-Advice)	Wrong study design
Williamson et al.[40]	2013	Modification of the school cafeteria environment can impact childhood nutrition. Results from the Wise Mind and LA Health studies	Wrong study design
Wolfenden et al.[41]	2014	A randomised controlled trial of an intervention to increase the implementation of a healthy canteen policy in Australian primary schools: study protocol	Appropriate data unavailable
Wolfenden et al.[42]	2019	Two-year follow-up of a randomised controlled trial to assess the sustainability of a school intervention to improve the implementation of a school-based nutrition policy	Outcomes not assessed
Wyse et al.[43]	2016	The potential of online canteens to deliver public health nutrition interventions to school communities	Appropriate data unavailable
Yoong et al.[44]	2016	CAFÉ: A multicomponent audit and feedback intervention to improve implementation of healthy food policy in primary school canteens: a randomised controlled trial	Outcomes not assessed

Figure S1 Components of school interventions included in the systematic review (1996-2023, n = 51).

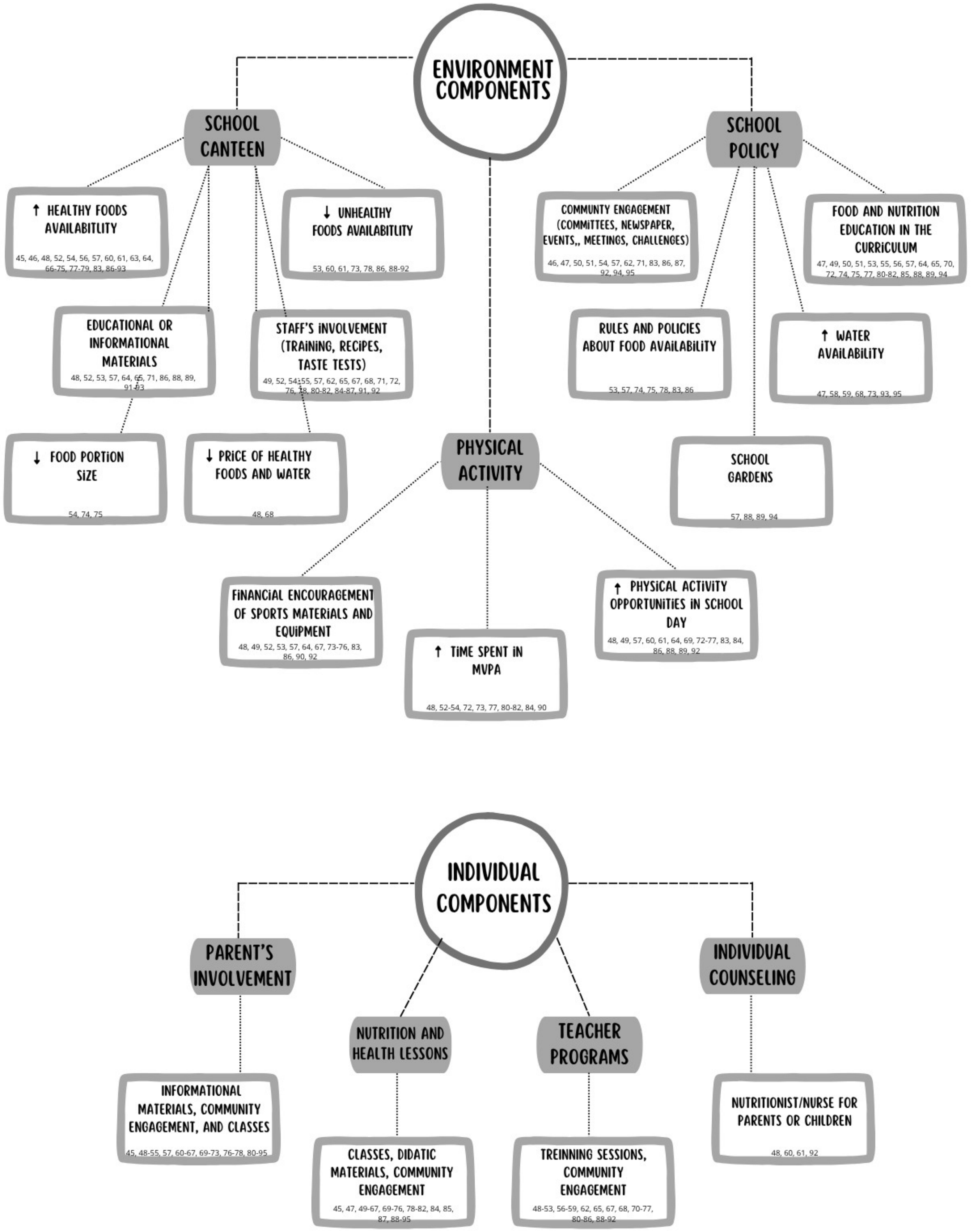

Figure S2 Conclusion summary of school interventions with the certainty of evidence (GRADE).

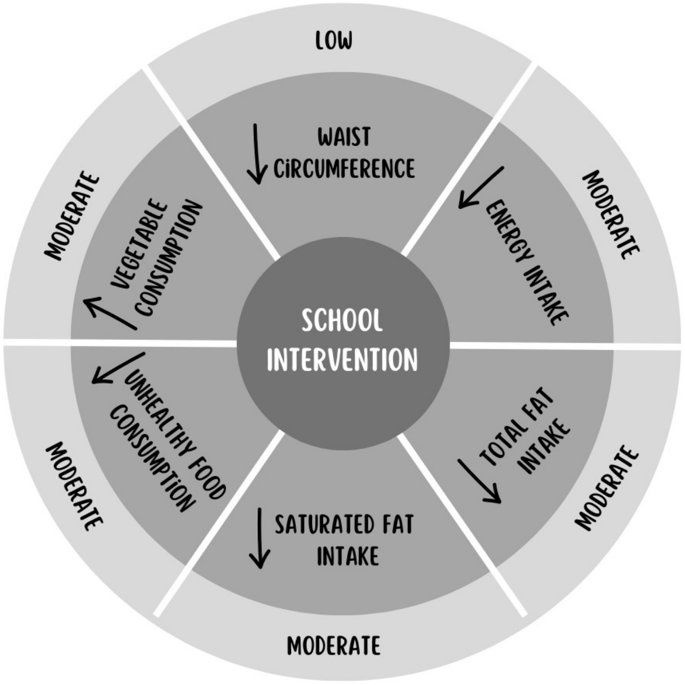

Figure S3 Sensitivity analysis excluding studies with high or serious risk of bias of the effect of intervention in the school food environment on body mass index (kg/m²). CI, confidence interval; MD, mean difference; SD, standard deviation.

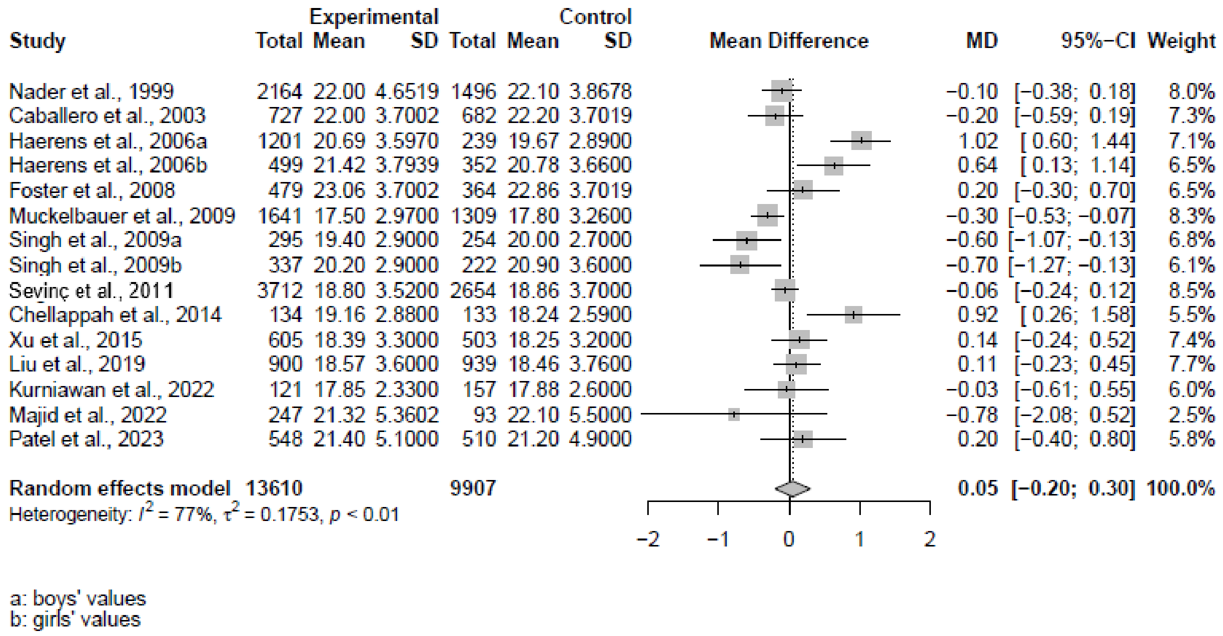

Figure S4 Sensitivity analysis excluding studies with high or serious risk of bias of the effect of intervention in the school food environment on body mass index (z-score). CI, confidence interval; MD, mean difference; SD, standard deviation.

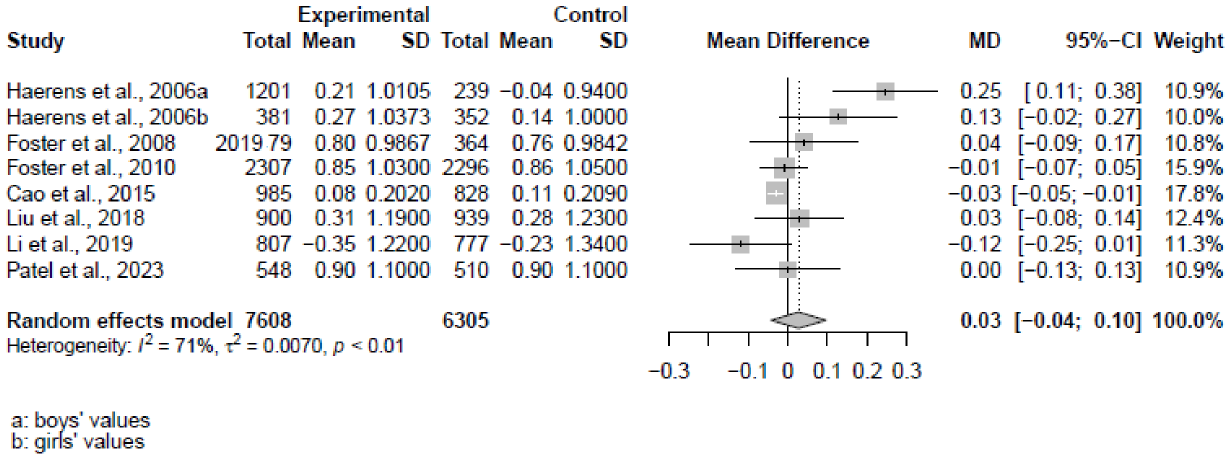

Figure S5 Sensitivity analysis excluding studies with high or serious risk of bias of the effect of intervention in the school food environment on waist circumference (cm). CI, confidence interval; MD, mean difference; SD, standard deviation.

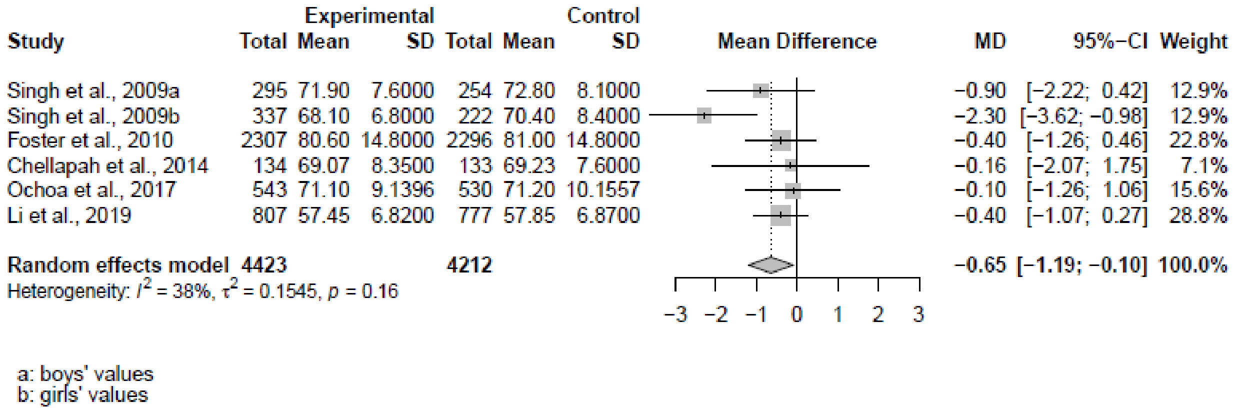

Figure S6 Sensitivity analysis excluding studies with high or serious risk of bias of the effect of intervention in the school food environment on body fat (%). CI, confidence interval; MD, mean difference; SD, standard deviation.

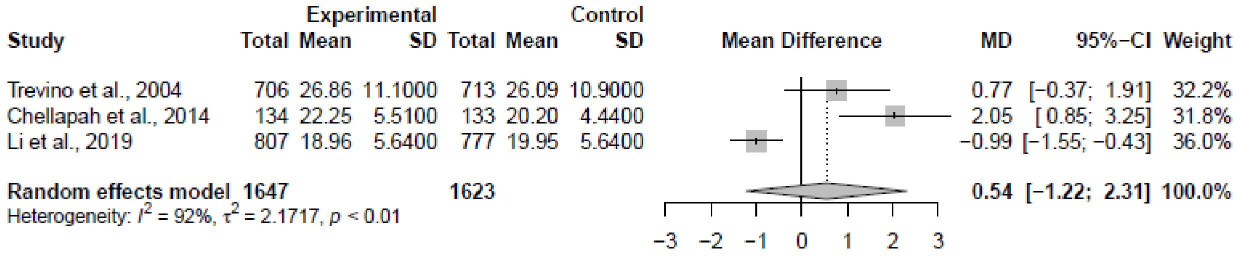

Figure S7 Risk of bias assessment according to the Cochrane Collaboration's tool (RoB 2.0) for randomized controlled trials.[96]

Alvirde-García et al., 2013	O	O	+	+	O	O
Amini et al., 2016	-	+	+	+	O	-
Bogart et al., 2016	-	O	+	+	O	-
Caballero et al., 2003	+	+	+	+	O	O
Cao et al., 2015	+	+	+	+	O	O
Chellappah et al., 2014	+	+	+	+	O	O
Coleman et al., 2012	-	O	+	+	O	-
Colín-Ramírez et al., 2009	+	+	+	+	O	O
Davis et al., 2021	+	O	+	+	+	O
Foster et al., 2008	-	O	+	+	O	-
Foster et al., 2010	+	+	+	+	+	+
Haerens et al., 2006	+	O	+	+	O	O
Herscovici et al., 2013	+	+	+	+	O	O
Kain et al., 2004	O	+	+	+	O	O
Levy et al., 2012	+	+	+	+	O	O
Li et al., 2019	+	+	+	+	+	+
Liu et al., 2019	+	+	+	+	O	O
Luepker et al., 1996	O	+	+	+	+	O
Marcus et al., 2009	O	-	-	+	O	-
Muckelbauer et al., 2009	O	+	+	+	O	O
Muckelbauer et al., 2009	+	+	+	+	O	O
Nader et al., 1999	+	+	+	+	+	+
Ochoa-Aviles et al., 2017	+	+	+	+	O	O
Ooi et al., 2021	+	+	+	+	+	+
Patel et al., 2023	+	+	+	+	+	+
Safdie et al., 2013	+	+	+	+	+	+

	Risk of bias arising from the randomization process	Risk of bias due to deviations from the intended	Risk of bias due to missing outcome data	Risk of bias in measurement of the outcome	Risk of bias in selection of the reported result	Overall bias
Sahota et al., 2001	-	O	+	-	O	-
Sallis et al., 2003	-	O	+	O	O	-
Sevinç et al., 2011	+	+	+	+	O	O
Singh et al., 2007	+	O	+	+	O	O
Singh et al., 2009	+	+	+	+	O	O
Singhal et al., 2011	-	O	+	+	O	-
Singhal et al., 2010	O	O	+	+	O	O
Treviño et al., 2004	+	+	+	+	O	O
Webber et al., 1996	+	+	+	+	O	O
Williamson et al., 2007	O	O	+	-	O	-
Williamson et al., 2012	+	+	+	+	+	+
Xu et al., 2014	O	+	+	+	O	O
Xu et al., 2015	+	+	+	+	O	O

Key

- + low risk of bias
- O some concerns
- - high risk of bias

Figure S8 Risk of bias assessment according to the Cochrane Collaboration's tool (ROBINS-I) for non-randomized intervention studies.[96]

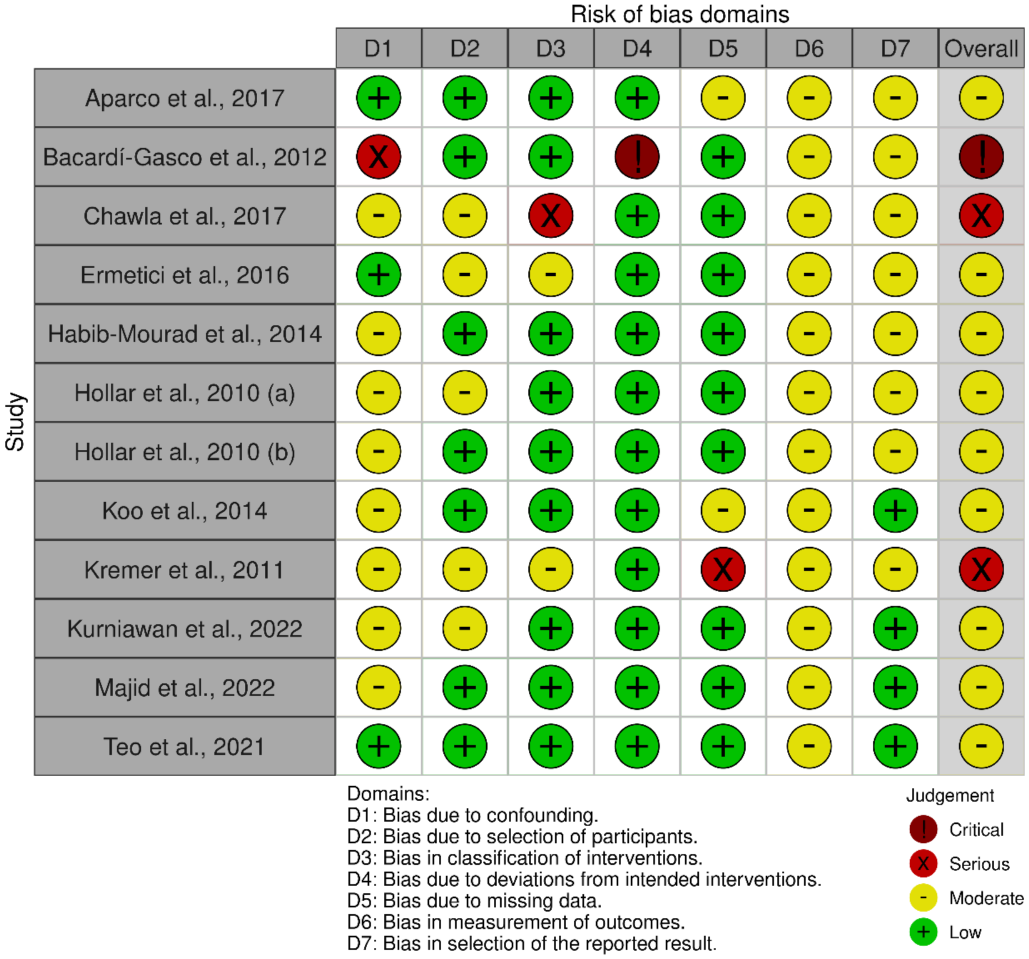

Table S5 Grading of Recommendations Assessment, Development and Evaluation (GRADE) assessment.

Participants (studies)	Risk of bias	Inconsistency	Indirectness	Imprecision	Publication bias	Overall certainty of evidence
			**Adiposity**			
**Body Mass Index (z-score)**						
29843 (15 CRCT 4 QE)	serious[a]	serious[b]	not serious	not serious	strongly suspected[c]	⊕◯◯◯ VERY LOW
**Body Mass Index (kg/m²)**						
38486 (21 cRCT 5 QE)	serious[a]	serious[b]	not serious	not serious	none	⊕⊕◯◯ LOW
**Waist Circumference (cm)**						
16593 (11 cRCT 4 QE)	serious[a]	not serious	not serious	not serious	strongly suspected[d]	⊕⊕◯◯ LOW
**Body fat (%)**						
11997 (8 cRCT 3 QE)	serious[a]	serious[e]	not serious	serious[f]	none	⊕◯◯◯ VERY LOW
			**Food consumption**			
**Fruit consumption (times/week or portions/day)**						
9481 (6 cRCT 5 QE)	serious[a]	not serious	not serious	not serious	none	⊕⊕⊕◯ MODERATE
**Vegetables consumption (times/week or portions/day)**						
10025 (6 cRCT 4 QE)	serious[a]	not serious	not serious	not serious	none	⊕⊕⊕◯ MODERATE
**Unhealthy food consumption (times/week or portions/day)**						
4054 (6 cRCT 3 QE)	serious[a]	not serious	not serious	not serious	none	⊕⊕⊕◯ MODERATE
**Sugar-sweetened beverages consumption (times/week or portions/day)**						
19122 (11 cRCT 5 QE)	serious[a]	not serious	not serious	not serious	none	⊕⊕⊕◯ MODERATE
**Energy intake (kcal or kJ)**						
9294 (10 cRCT 1 QE)	serious[a]	not serious	not serious	not serious	none	⊕⊕⊕◯ MODERATE
**Total fat intake (grams/day or % kcal/day)**						
9210 (10 cRCT 1 QE)	serious[a]	not serious	not serious	not serious	none	⊕⊕⊕◯ MODERATE
**Saturated fat intake (% kcal/day)**						
5036 (5 cRCT)	serious[a]	not serious	not serious	not serious	none	⊕⊕⊕◯ MODERATE

[a] In the overall risk of bias assessment, most studies rated as presenting some concerns or a high risk of bias.

[b] The meta-analysis showed high heterogeneity (elevated I^2), with overlapping confidence intervals across studies but effect estimates in different directions. Additionally, the studies included in the narrative synthesis presented conflicting results, with some indicating a benefit of the intervention and others showing no effect.

[c] Visual inspection of the funnel plot suggested marked asymmetry, supported by Egger's test, indicating a potential risk of publication bias.

[d] The majority of studies reported positive results, while only a few studies showed null or negative effects.

[e] The meta-analysis showed high heterogeneity (elevated I^2), with overlapping confidence intervals across studies but effect estimates in different directions.

[f] Downgraded for imprecision due to wide confidence intervals including no effect.

[g] Inconsistent results across studies, with divergent effect directions (benefit vs. no effect), and no possibility to explore heterogeneity.

Figure S9a Funnel plot for publication bias assessment of studies included in the meta-analysis for body mass index z-score.

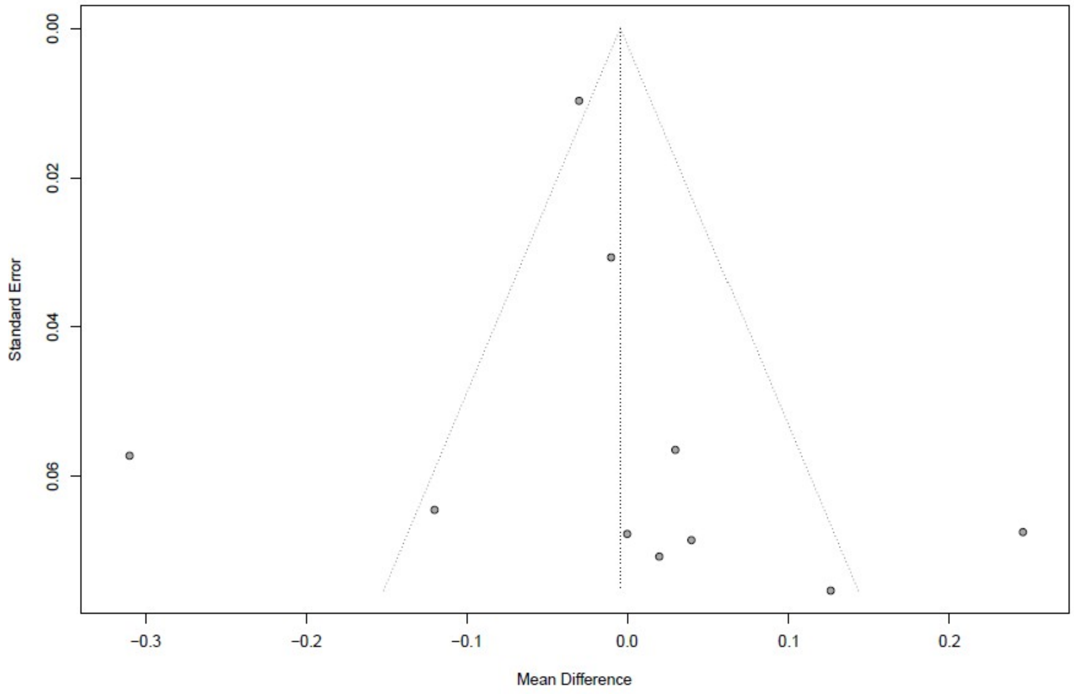

Figure S9b Funnel plot with Trim and Fill adjustment for the body mass index z-score meta-analysis.

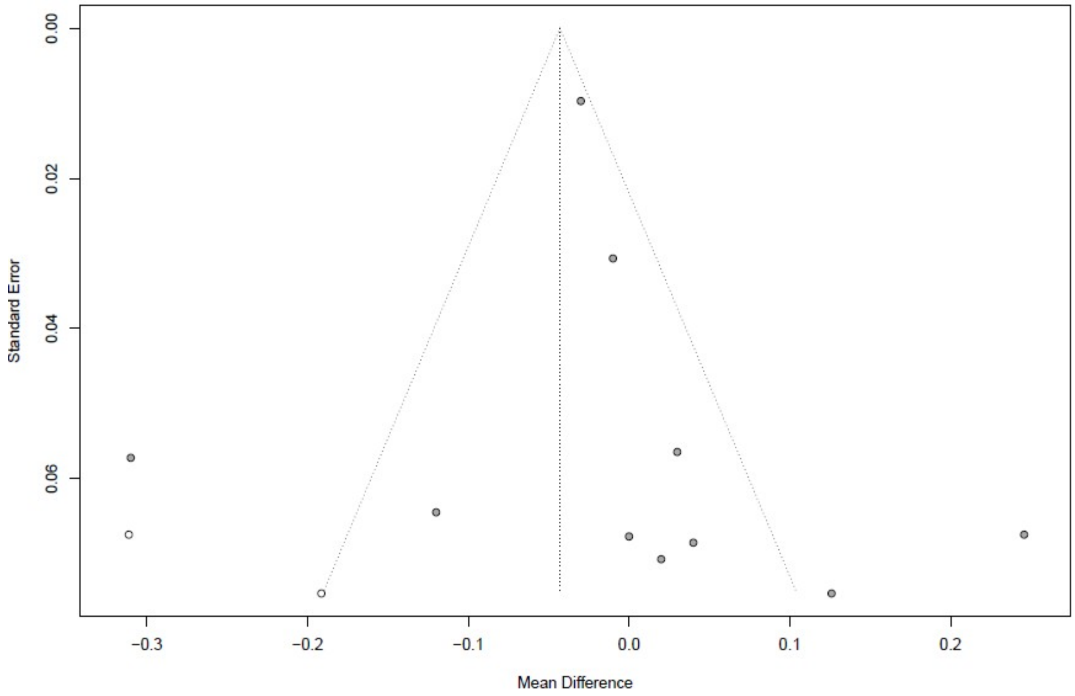

Figure S10 Funnel plot for publication bias assessment of studies included in the meta-analysis for body mass index (kg/m²).

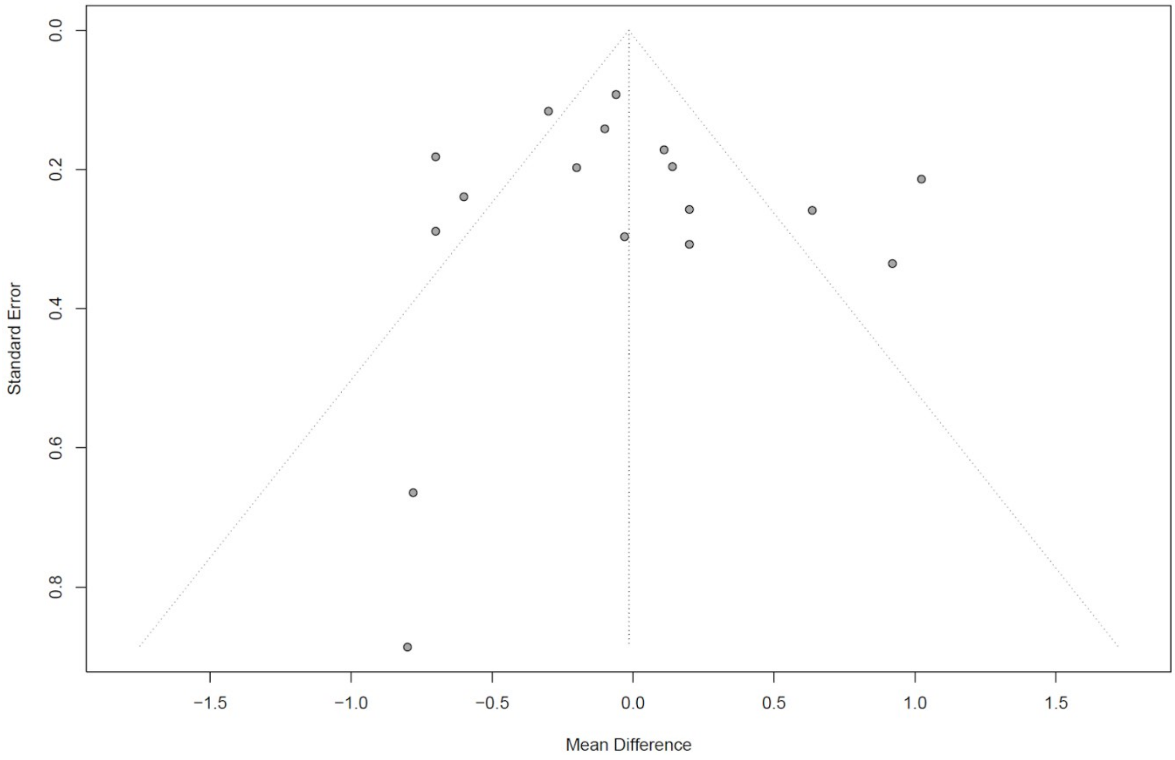

References

1. Page MJ, McKenzie JE, Bossuyt PM, Boutron I, Hoffmann TC, Mulrow CD, et al. 2021. The PRISMA 2020 statement: an updated guideline for reporting systematic reviews. BMJ 372:n71. PMID: 33782057, https://doi.org/10.1136/bmj .n71.
2. Almughamisi M, O'Keeffe M, Harding S. 2020. Promoting healthy school food environments in Jeddah, Saudi Arabia: an adolescent led canteen intervention appeals care providers .West Indian Med J 68(SUPPL 1):38. https://www.cochranelibrary.com/central/doi/10.1002/central/CN-02096323/full.
3. Andrade S, Lachat C, Ochoa-Aviles A, Verstraeten R, Huybregts L, Roberfroid D, et al. 2014. A school-based intervention improves physical fitness in Ecuadorian adolescents: a cluster-randomized controlled trial. Int J Behav Nutr Phys Act. 11:153, PMID: 25490946, https://doi.org/10.1186/s12966-014-0153-5.
4. Barbosa Filho VC, Bandeira ADS, Minatto G, Linard JG, Silva JAD, Costa RMD, et al. Effect of a Multicomponent Intervention on Lifestyle Factors among Brazilian Adolescents from Low Human Development Index Areas: A Cluster-Randomized Controlled Trial. Int J Environ Res Public Health 18;16(2):267. PMID: 30669291, https://doi.org/10.3390/ijerph16020267.
5. Berenson GS. 2010. Cardiovascular health promotion for children: a model for a Parish (County)-wide program (implementation and preliminary results). Prev Cardiol. 13(1):23-8 PMID: 20021623, https://doi.org/10.1111/j.1751-7141.2009.00049.x.
6. Birnbaum AS, Lytle LA, Story M, Perry CL, Murray DM. 2002. Are differences in exposure to a multicomponent school-based intervention associated with varying dietary outcomes in adolescents? Health Educ Behav 29(4):427-43. PMID: 12137237, https://doi.org/10.1177/109019810202900404.
7. Coleman KJ, Shordon M, Caparosa SL, Pomichowski ME, Dzewaltowski DA. 2012. The healthy options for nutrition environments in schools (Healthy ONES) group randomized trial: using implementation models to change nutrition policy and environments in low income schools. Int J Behav Nutr Phys Act. 27;9:80. PMID: 22734945, https://doi.org/10.1186/1479-5868-9-80.
8. Damsgaard CT, Dalskov SM, Petersen RA, Sørensen LB, Mølgaard C, Biltoft-Jensen A, et al. 2012. Design of the OPUS School Meal Study: a randomised controlled trial assessing the impact of serving school meals based on the New Nordic Diet. Scand J Public Health. 2012 Dec;40(8):693-703. PMID: 23108477, https://doi.org/10.1177/1403494812463173.
9. Davee AM, Blum JE, Devore RL, Beaudoin CM, Kaley LA, Leiter JL, et al. 2005. The vending and à la carte policy intervention in Maine public high schools. Prev Chronic Dis. 2005 Nov;2 Spec no(Spec No):A14. PMID: 16263047, https://www.cdc.gov/pcd/issues/2005/nov/05_0076.htm.

10. Florence W, Ochola S, Irene O. 2020. Physical and Nutrition Education Intervention Improves Body Weight Status of Adolescents' in Uasin Gishu County, Kenya: A Cluster-Randomized Controlled Trial. Curr Res Nutr Food Sci 8(1). http://dx.doi.org/10.12944/CRNFSJ.8.1.30.

11. Foster G, Hirst K. 2007. Middle-School Based Primary Prevention Trial of Type 2 Diabetes. National Institute of Diabetes and Digestive and Kidney Diseases (NIDDK). https://www.clinicaltrials.gov/study/NCT00458029.

12. French SA, Story M, Jeffery RW, Snyder P, Eisenberg M, Sidebottom A, et al. 1997. Pricing strategy to promote fruit and vegetable purchase in high school cafeterias. J Am Diet Assoc. 97(9):1008-10. PMID: 9284880, https://doi.org/10.1016/S0002-8223(97)00242-3.

13. French SA, Story M, Fulkerson JA, Hannan P. 2004. An environmental intervention to promote lower-fat food choices in secondary schools: outcomes of the TACOS Study. Am J Public Health. 94(9):1507-12. PMID: 15333303, https://doi.org/10.2105/ajph.94.9.1507.

14. Fu YCA, To KC, Tao WY, Kwan KMA, Lee YH, Fung YKA, et al. 2019. School accreditation scheme reduces childhood obesity in Hong Kong [published correction appears in Glob Health Promot. 2021 Sep;28(3):NP1]. Glob Health Promot. 26(4):70-78. PMID: 29809101, https://doi.org/10.1177/1757975918764318.

15. Gallotta MC, Iazzoni S, Emerenziani GP, Meucci M, Migliaccio S, Guidetti L, et al. 2016. Effects of combined physical education and nutritional programs on schoolchildren's healthy habits. PeerJ. 11;4:e1880. PMID: 27077004, https://doi.org/10.7717/peerj.1880.

16. Haerens L, Deforche B, Maes L, Cardon G, Stevens V, De Bourdeaudhuij I. 2006. Evaluation of a 2-year physical activity and healthy eating intervention in middle school children. Health Educ Res. 21(6):911-21. PMID: 17032704, https://doi.org/10.1093/her/cyl115.

17. Lane HG, Deitch R, Wang Y, Black MM, Dunton GF, Aldoory L, et al. 2018. Wellness Champions for Change," a multi-level intervention to improve school-level implementation of local wellness policies: Study protocol for a cluster randomized trial. Contemp. Clin. Trials 75:29-39. https://doi.org/10.1016/j.cct.2018.10.008.

18. Hoppu U, Lehtisalo J, Kujala J, Keso T, Garam S, Tapanainen H, et al. 2010. The diet of adolescents can be improved by school intervention. Public Health Nutr. 13(6A):973-9. PMID: 20513268, https://doi.org/10.1017/S1368980010001163.

19. Husain Y. 2020. Three years follow up (cohort) study via population based intervention on adolescent and childhood obesity and overweight at schools setting. Special Issue: Abstracts from the European and International Congress on Obesity. 21(S1):e13118. https://doi.org/10.1111/obr.13118.

20. Leme ACB, Baranowski T, Thompson D, Nicklas T, Philippi ST. 2018. Sustained impact of the "Healthy Habits, Healthy Girls - Brazil" school-based randomized controlled trial for adolescents living in low-income communities. Prev Med Rep. 10:346-352. PMID: 29868390, https://doi.org/10.1016/j.pmedr.2018.04.013.

21. Li XH, Lin S, Guo H, Huang Y, Wu L, Zhang Z, et al. 2014. Effectiveness of a school-based physical activity intervention on obesity in school children: a nonrandomized controlled trial. BMC Public Health.14:1282. PMID: 25510313, https://doi.org/10.1186/1471-2458-14-1282.

22. Lytle LA. 1998. Lessons from the Child and Adolescent Trial for Cardiovascular Health (CATCH): interventions with children. Curr Opin Lipidol. 9(1):29-33. PMID: 9502332, https://doi.org/10.1097/00041433-199802000-00007.

23. Lytle LA, Murray DM, Perry CL, Story M, Birnbaum AS, Kubik MY, et al. 2004. School-based approaches to affect adolescents' diets: results from the TEENS study. Health Educ Behav. 31(2):270-87. PMID: 15090126, https://doi.org/10.1177/1090198103260635.

24. Majid HA. 2018. A school-based study to improve healthy eating habits amongst Malaysian teenagers. ISRCTN registry. https://doi.org/10.1186/ISRCTN89649533.

25. Moreno GD, Schmidt LA, Ritchie LD, McCulloch CE, Cabana MD, Brindis CD, Green LW, Altman EA, Patel AI. 2021. A cluster-randomized controlled trial of an elementary school drinking water access and promotion intervention: Rationale, study design, and protocol. Contemp Clin Trials. 101:106255. PMID: 33370616, https://doi.org/10.1016/j.cct.2020.106255.

26. Nik Husain NR, Manan W, Jamil NIN, Hanafi NNN, Rahman RA. 2018. Evaluating the implementation of a canteen-based food nutrition intervention among schoolchildren: a prospective intervention study. JUMMEC 2018:21(1). https://www.researchgate.net/publication/327051942_Evaluating_the_Implementation_of_a_Canteen_Based_Food_Nutrition_Intervention_among_Schoolchildren_A_Prospective_Intervention_Study_JUMMEC_211_21-27.

27. Ochoa A, Ochoa AA, Andrade TDS, Verstraeten R, Huybregts L, Lachat C, et al. 2017. Effect of the school-based health promotion intervention activital on dietary intake and waist circumference: A cluster randomized controlled trial. Annals of Nutrition and Metabolism 71(Supplement):1272-1273. https://doi.org/10.1159/000480486.

28. Olsen NJ, Ängquist L, Frederiksen P, Lykke Mortensen E, Heitmann BL. 2021. Primary prevention of fat and weight gain among obesity susceptible healthy weight preschool children. Main results from the "Healthy Start" randomized controlled intervention. Pediatr Obes. 16(4):e12736. PMID: 33021348, https://doi.org/10.1111/ijpo.12736.

29. Perry CL, Bishop DB, Taylor GL, Davis M, Story M, Gray C, et al. 2004. A randomized school trial of environmental strategies to encourage fruit and vegetable consumption among

children. Health Educ Behav. 31(1):65-76. PMID: 14768658, https://doi.org/10.1177/1090198103255530.
30. Pramesthi IL, Wangge G, Ananda AJN, Ermayani E, Iswari DN. 2019. Intervention of the nutrition goes to school program for adolescents in Malang district, East Java: Baseline report. Annals of Nutrition and Metabolism 75(3):204. https://doi.org/10.1159/000501751.
31. Ramos MJJ. 2019. Educación para la salud. Nutrición y gastronomía en las ciudades autónomas de Melilla y Ceuta [Education for health. Nutrition and gastronomy in the Autonomous Cities of Melilla and Ceuta]. Nutr Hosp. 36(Spec No1):135-138. https://doi:10.20960/nh.02728.
32. Rerksuppaphol L, Rerksuppaphol S. 2017. Internet Based Obesity Prevention Program for Thai School Children- A Randomized Control Trial. J Clin Diagn Res. 11(3):SC07-SC11. PMID: 28511471, https://doi.org/10.7860/JCDR/2017/21423.93 68.
33. Schwartz AE, Leardo M, Aneja S, Elbel B. 2016. Effect of a School-Based Water Intervention on Child Body Mass Index and Obesity. JAMA Pediatr. 170(3):220-6. PMID: 26784336, https://doi.org/10.1001/jamapediatrics.2015.3778.
34. Serra-Paya N, Ensenyat A, Castro-Viñuales I, Real J, Sinfreu-Bergués X, Zapata A, et al. Effectiveness of a Multi-Component Intervention for Overweight and Obese Children (Nereu Program): A Randomized Controlled Trial. PLoS One. 10(12):e0144502. PMID: 26658988, https://doi.org/10.1371/journal.pone.01445 02.
35. Sichieri R, Paula Trotte A, de Souza RA, Veiga GV. 2009. School randomised trial on prevention of excessive weight gain by discouraging students from drinking sodas. Public Health Nutr. 2:197-202. PMID: 18559131. https://doi.org/10.1017 /S1368980008002644
36. Singh AS, Chin A Paw MJ, Kremers SP, Visscher TL, Brug J, van Mechelen W. 2006. Design of the Dutch Obesity Intervention in Teenagers (NRG-DOiT): systematic development, implementation and evaluation of a school-based intervention aimed at the prevention of excessive weight gain in adolescents. BMC Public Health. 6:304. PMID: 17173701, https://doi.org/10.1186/1471-2458-6-304.
37. Schuh DS. 2015. Program for health promotion in schools of public elementary school in the state of Rio Grande do Sul. ICTRP registry. https://www.cochranelibrary.com/central/doi/10.1002/central/CN-01825893/full.
38. Teo CH, Chin YS, Lim PY, Masrom SAH, Shariff ZM. 2019. School-based intervention that integrates nutrition education and supportive healthy school food environment among Malaysian primary school children: a study protocol. BMC Public Health. 19(1):1427. PMID: 31666034, https://doi.org/10.1186/s12889-019-7708-y.
39. Turnin MC, Buisson JC, Ahluwalia N, Cazals L, Bolzonella-Pene C, Fouquet-Martineau C, et al. 2016. Effect of Nutritional Intervention on Food Choices of French Students in Middle School

Cafeterias, Using an Interactive Educational Software Program (Nutri-Advice). J Nutr Educ Behav. 48(2):131-7.e1. PMID: 26548405, https://doi.org/10.1016/j.jneb.2015.09.011.

40. Williamson DA, Han H, Johnson WD, Martin CK, Newton RL Jr. 2013. Modification of the school cafeteria environment can impact childhood nutrition. Results from the Wise Mind and LA Health studies. Appetite. 61(1):77-84. PMID: 23154216, https://doi.org/10.1016/j.appet.2012.11.002.

41. Wolfenden L, Nathan N, Williams CM, Delaney T, Reilly KL, Freund M, et al. 2014. A randomised controlled trial of an intervention to increase the implementation of a healthy canteen policy in Australian primary schools: study protocol. Implement Sci. 2014 Oct 11;9:147. PMID: 25300221, https://doi.org/10.1186/s13012-014-0147-3.

42. Wolfenden L, Nathan N, Reilly K, Delaney T, Janssen LM, Reynolds R, et al. 2019. Two-year follow-up of a randomised controlled trial to assess the sustainability of a school intervention to improve the implementation of a school-based nutrition policy. Health Promot J Austr. 30(Suppl 1):26-33. PMID: 30805958, https://doi.org/10.1 002/hpja.238.

43. Wyse R, Yoong SL, Dodds P, Campbell L, Delaney T, Nathan N, et al. 2016. The potential of online canteens to deliver public health nutrition interventions to school communities. Int. J. Behav. Med. 23(Suppl 1):S67. https://doi.org/10.1007/s12529-016-9586-3.

44. Yoong SL, Nathan N, Wolfenden L, Wiggers J, Reilly K, Oldmeadow C, et al. 2016. CAFÉ: a multicomponent audit and feedback intervention to improve implementation of healthy food policy in primary school canteens: a randomised controlled trial. Int J Behav Nutr Phys Act. 13(1):126. PMID: 27919261, https://doi.org/10.1186/s12966-016-0453-z.

45. Herscovici RC, Kovalskys I, De Gregorio MJ. 2013. Gender differences and a school-based obesity prevention program in Argentina: a randomized trial. Rev Panam Salud Publica 34(2):75-82. PMID: 24096971, https://iris.paho.org/handle/10665.2/9150.

46. Chellappah J, Tonkin A, Gregg MED, Reid C. 2015. A Randomized Controlled Trial of Effects of Fruit Intake on Cardiovascular Disease Risk Factors in Children (FIST Study). Infant, Child, & Adolescent Nutrition: ICAN 7(1):15-23. https://doi.org/10.1177/19414064145539.

47. Ooi JY, Wolfenden L, Yoong SL, Janssen LM, Reilly K, Nathan N, et al. 2021. A trial of a six-month sugar-sweetened beverage intervention in secondary schools from a socio-economically disadvantaged region in Australia. Aust N Z J Public Health 45(6):599-607 PMID: 34761854, https://doi.org/10.1111/1753-6405.13159.

48. Haerens L, Deforche B, Maes L, Stevens V, Cardon G, De Bourdeaudhuij I. 2006. Body mass effects of a physical activity and healthy food intervention in middle schools. Obesity (Silver Spring) 14(5):847-854. PMID: 16855194, https://doi.org /10.1038/oby.2006.98.

49. Kain J, Uauy R, Albala, Vio F, Cerda R, Leyton B. 2004. School-based obesity prevention in Chilean primary school children: methodology and evaluation of a controlled study. Int J Obes Relat Metab Disord. 28(4):483-93. PMID: 14993915, https://doi.org/10.1038/sj.ijo.0802611.

50. Xu F, Wang X, Ware RS, Tse LA, Wang Z, Hong X, et al. 2014. A school-based comprehensive lifestyle intervention among Chinese kids against Obesity (CLICK-Obesity) in Nanjing City, China: the baseline data. Asia Pac J Clin Nutr. 23(1):48-54. PMID: 24561972, https://doi.org/10.6133/apjcn.2014.23.1.04.

51. Xu F, Ware RS, Leslie E, Tse LA, Wang Z, Li J, et al. 2015. Effectiveness of a Randomized Controlled Lifestyle Intervention to Prevent Obesity among Chinese Primary School Students: CLICK-Obesity Study. PLoS One 28;10(10):e0141421. PMID: 26510135, https://doi.org/10.1371/journal.pone.0141421.

52. Cao ZJ, Wang SM, Chen Y. 2015. A randomized trial of multiple interventions for childhood obesity in China. Am J Prev Med. 48(5):552-60. PMID: 25891054, https://doi.org/10.1016/j.amepre.2014.12.014.

53. Liu Z, Li Q, Maddison R, Ni Mhurchu C, Jiang Y, Wei DM, et al. 2019. A School-Based Comprehensive Intervention for Childhood Obesity in China: A Cluster Randomized Controlled Trial. Child Obes. 15(2):105-115. PMID: 30565955, https://doi.org/10.1089/chi.2018.0251.

54. Li B, Pallan M, Liu WJ, Hemming K, Frew E, Lin R, et al. 2019. The CHIRPY DRAGON intervention in preventing obesity in Chinese primary-school-aged children: A cluster-randomised controlled trial. PLoS Med. 16(11):e1002971. PMID: 31770371, https://doi.org/10.1371/journal.pmed.10029 71.

55. Ochoa-Avilés A, Verstraeten R, Huybregts L, Andrade S, Van Camp J, Donoso S, et al. 2017. A school-based intervention improved dietary intake outcomes and reduced waist circumference in adolescents: a cluster randomized controlled trial. Nutr J.16(1):79. PMID: 29228946, https://doi.org/10.1186/s12937-017-0299-5.

56. Sahota P, Rudolf MC, Dixey R, Hill AJ, Barth JH, Cade J. 2001. Randomised controlled trial of primary school based intervention to reduce risk factors for obesity. BMJ 323(7320):1029-32. PMID: 11691759, https://doi.org/10.1136/bmj. 323.7320.1029.

57. Kremer P, Waqa G, Vanualailai N, Schultz JT, Roberts G, Moodie M, et al. 2011. Reducing unhealthy weight gain in Fijian adolescents: results of the Healthy Youth Healthy Communities study. Obes Rev. 12 Suppl 2:29-40. PMID: 22008557, https://doi.org/10.1111/j.1467-789X.2011.00912.x.

58. Muckelbauer R, Libuda L, Clausen K, Toschke AM, Reinehr T, Kersting M. 2009. Promotion and provision of drinking water in schools for overweight prevention: randomized,

controlled cluster trial. Pediatrics 123(4):e661-7. PMID: 19336356, https://doi.org/10.1542/peds.2008-2186.
59. Muckelbauer R, Libuda L, Clausen K, Reinehr T, Kersting M. 2009. A simple dietary intervention in the school setting decreased incidence of overweight in children. Obes Facts. 2(5):282-5. PMID: 20057194, https://doi.org/10.1159/0002 29783.
60. Singhal N, Misra A, Shah P, Gulati S. 2010. Effects of controlled school-based multi-component model of nutrition and lifestyle interventions on behavior modification, anthropometry and metabolic risk profile of urban Asian Indian adolescents in North India. Eur J Clin Nutr. 64(4):364-73. PMID: 20087379, https://doi.org/10.1038/ejcn.2009.150.
61. Singhal N, Misra A, Shah P, Gulati S, Bhatt S, Sharma S, et al. 2011. Impact of intensive school-based nutrition education and lifestyle interventions on insulin resistance, β-cell function, disposition index, and subclinical inflammation among Asian Indian adolescents: a controlled intervention study. Metab Syndr Relat Disord. 9(2):143-50. PMID: 21118028, https://doi.org/10.1089/met.2010.0094.
62. Kurniawan F, Prabandari YS, Ismail D, Dewi FT. 2022. Effectiveness of school-based obesity prevention programme among elementary school children in Jakarta. Mal J Nutr. 28(1):097-106. https://doi.org/10.31246/mjn-2020-0101.
63. Amini M, Djazayery A, Majdzadeh R, Taghdisi MH, Sadrzadeh-Yeganeh H, Abdollahi Z, et al. 2016. A School-Based Intervention to Reduce Excess Weight in Overweight and Obese Primary School Students. Biol Res Nurs. 18(5):531-40. PMID: 27358261, https://doi.org/10.1177/1099800416654261.
64. Ermetici F, Zelaschi RF, Briganti S, Dozio E, Gaeta M, Ambrogi F, et al. 2016. Association between a school-based intervention and adiposity outcomes in adolescents: The Italian "EAT" project. Obesity (Silver Spring) 24(3):687-95. PMID: 26833570, https://doi.org/10.1002/oby.21365.
65. Habib-Mourad C, Ghandour LA, Moore HJ, Nabhani-Zeidan M, Adetayo K, Hwalla N, et al. 2014. Promoting healthy eating and physical activity among school children: findings from Health-E-PALS, the first pilot intervention from Lebanon. BMC Public Health 14:940. https://doi.org/10.1186/1471-2458-14-940.
66. Koo HC, Poh BK, Abd Talib R. 2018. The GReat-Child™ Trial: A Quasi-Experimental Intervention on Whole Grains with Healthy Balanced Diet to Manage Childhood Obesity in Kuala Lumpur, Malaysia. Nutrients 10(2):156. PMID: 29385769, https://doi.org/10.3390/nu10020156.
67. Teo CH, Chin YS, Lim PY, Masrom SAH, Shariff ZM. 2021. Impacts of a School-Based Intervention That Incorporates Nutrition Education and a Supportive Healthy School Canteen Environment among Primary School Children in Malaysia. Nutrients May 18;13(5):1712. PMID: 34070053, https://doi.org/10.339 0/nu13051712.

68. Majid HA, Ng AK, Dahlui M, Mohammadi S, Mohamed MNAB, Su TT, et al. 2022. Outcome Evaluation on Impact of the Nutrition Intervention among Adolescents: A Feasibility, Randomised Control Study from Myheart Beat (Malaysian Health and Adolescents Longitudinal Research Team-Behavioural Epidemiology and Trial). Nutrients 14(13):2733. PMID: 35807911, https://doi.org/10.3390/nu14132733.
69. Colín-Ramírez E, Castillo-Martínez L, Orea-Tejeda A, Vergara A, Villa AR. 2009. Efecto de una intervención escolar basada en actividad física y dieta para la prevención de factores de riesgo cardiovascular (RESCATE) en niños mexicanos de 8 a 10 años. Rev Esp Nutr Comunitaria 15(2):71-80. https://www.renc.es/imagenes/auxiliar/files/2009-2._Eloisa.pdf.
70. Bacardí-Gascon M, Pérez-Morales ME, Jiménez-Cruz A. 2012. A six month randomized school intervention and an 18-month follow-up intervention to prevent childhood obesity in Mexican elementary schools. Nutr Hosp. 27(3):755-62. PMID: 23114940, https://doi.org/10.3305/nh.2012.27.3.5756.
71. Levy TS, Ruán CM, Castellanos CA, Coronel AS, Aguilar AJ, Humarán IMG. 2012. Effectiveness of a diet and physical activity promotion strategy on the prevention of obesity in Mexican school children. BMC Public Health 12:152. PMID: 22381137, https://doi.org/10.1186/1471-2458-12-152.
72. Alvirde-García U, Rodríguez-Guerrero AJ, Henao-Morán S, Gómez-Pérez FJ, Aguilar-Salinas CA. 2013. Resultados de un programa comunitario de intervención en el estilo de vida en niños [Results of a community-based life style intervention program for children]. Salud Pública de Mex 55(Supl. 3):406-414. PMID: 24643489. https://www.scielo.org.mx/scielo.php?script=sci_arttext&pid =S0036-36342013000900007.
73. Safdie M, Jennings-Aburto N, Lévesque L, Janssen I, Campirano-Núñez F, López-Olmedo N, et al. 2013. Impact of a school-based intervention program on obesity risk factors in Mexican children. Salud Publica Mex. 55(Suppl 3):374-87. PMID: 24643486, https://doi.org/10.21149/spm.v55s3.5138.
74. Singh AS, Chin A Paw MJ, Brug J, van Mechelen W. 2007. Short-term effects of school-based weight gain prevention among adolescents. Arch Pediatr Adolesc Med. 161(6):565-71. PMID: 17548761, https://doi.org/10.1001/archpedi.161.6.5 65.
75. Singh AS, Chin A Paw MJ, Brug J, van Mechelen W. 2009. Dutch obesity intervention in teenagers: effectiveness of a school-based program on body composition and behavior. Arch Pediatr Adolesc Med.163(4):309-17. PMID: 19349559, https://doi.org/10.1001/archpediatrics.2009.2.
76. Aparco JP, Bautista-Olórtegui W, Pillaca J. 2017. Evaluación del impacto de la intervención educativa-motivacional "Como Jugando" para prevenir la obesidad en escolares del Cercado de Lima: resultados al primer año [Impact evaluation of educational-motivational intervention "Como

Jugando" to prevent obesity in school children of Cercado de Lima: results in the first year]. Rev Peru Med Exp Salud Publica 34(3):386-394. PMID: 29267762, https://doi.org/10.17843/rpmesp.2017.343.2472.

77. Marcus C, Nyberg G, Nordenfelt A, Karpmyr M, Kowalski J, Ekelund U. 2009. A 4-year, cluster-randomized, controlled childhood obesity prevention study: STOPP. Int J Obes (Lond). 33(4):408-17. PMID: 19290010, https://doi.org/10.1038/ijo.2009.38.

78. Chawla N, Panza A, Sirikulchayanonta C, Kumar R, Taneepanichskul S. 2017. Effectiveness Of A School-Based Multicomponent Intervention On Nutritional Status Among Primary School Children In Bangkok, Thailand. J Ayub Med Coll Abbottabad 29(1):13-20. PMID: 28712165, https://jamc.ayubmed.edu.pk/jamc/index.php/jamc/article/view/1213/856.

79. Sevinç Ö, Bozkurt AI, Gündoğdu M, Aslan ÜB, Ağbuğa B, Aslan Ş, et al. 2011. Evaluation of the effectiveness of an intervention program on preventing childhood obesity in Denizli, Turkey. Turk J Med Sci 41(6):1097-1105. https://doi.org/10.3906/sag-1009-1179.

80. Luepker RV, Perry CL, McKinlay SM, Nader PR, Parcel GS, Stone EJ, et al. 1996. Outcomes of a field trial to improve children's dietary patterns and physical activity. The Child and Adolescent Trial for Cardiovascular Health. CATCH collaborative group. JAMA 275(10):768-76. PMID: 8598593, https://doi.org/10.1001/jama.1996.03530340032026.

81. Webber LS, Osganian SK, Feldman HA, Wu M, McKenzie TL, Nichaman M, et al. 1996. Cardiovascular risk factors among children after a 2 1/2-year intervention-The CATCH Study. Prev Med. 25(4):432-41. PMID: 8818067, https://doi.org/10.1006/pmed.1996.0075.

82. Nader PR, Stone EJ, Lytle LA, Perry CL, Osganian SK, Kelder S, et al. 1999. Three-year maintenance of improved diet and physical activity: the CATCH cohort. Child and Adolescent Trial for Cardiovascular Health. Arch Pediatr Adolesc Med. 153(7):695-704. PMID: 10401802, https://doi.org/10.1001/archpe di.153.7.695.

83. Sallis JF, McKenzie TL, Conway TL, Elder JP, Prochaska JJ, Brown M, et al. 2003. Environmental interventions for eating and physical activity: a randomized controlled trial in middle schools. Am J Prev Med. 24(3):209-17. PMID: 12657338, https://doi.org/10.1016/s0749-3797(02)00646-3.

84. Caballero B, Clay T, Davis SM, Ethelbah B, Rock BH, Lohman T, et al. 2003. Pathways: a school-based, randomized controlled trial for the prevention of obesity in American Indian schoolchildren. Am J Clin Nutr. 78(5):1030-8. PMID: 14594792, https://doi.org/10.1093/ajcn/78.5.1030.

85. Treviño RP, Yin Z, Hernandez A, Hale DE, Garcia OA, Mobley C. 2004. Impact of the Bienestar school-based diabetes mellitus prevention program on fasting capillary glucose levels: a randomized controlled trial [published correction appears in Arch Pediatr Adolesc Med. 2005 Apr;

159(4):341]. Arch Pediatr Adolesc Med. 158(9):911-917. PMID: 15351759, https://doi.org/10.1001/archpe di.158.9.911.

86. Williamson DA, Copeland AL, Anton SD, Champagne C, Han H, Lewis L, et al. 2007. Wise Mind project: a school-based environmental approach for preventing weight gain in children. Obesity (Silver Spring) 15(4):906-17. PMID: 17426326, https://doi.org/10.1038/oby.2007.597.

87. Foster GD, Sherman S, Borradaile KE, Grundy KM, Vander Veur SS, Nachmani J, et al. 2008. A policy-based school intervention to prevent overweight and obesity. Pediatrics 121(4):e794-802. PMID: 18381508, https://doi.org/10.1542/pe ds.2007-1365.

88. Hollar D, Lombardo M, Lopez-Mitnik G, Hollar TL, Almon M, Agatston AS, et al. 2010 (a). Effective multi-level, multi-sector, school-based obesity prevention programming improves weight, blood pressure, and academic performance, especially among low-income, minority children. J Health Care Poor Underserved 21(2 Suppl):93-108. PMID: 20453379, https://doi.org/10.1353/hpu.0.0304.

89. Hollar D, Messiah SE, Lopez-Mitnik G, Hollar TL, Almon M, Agatston AS. 2010 (b). Healthier options for public schoolchildren program improves weight and blood pressure in 6- to 13-year-olds. J Am Diet Assoc. 110(2):261-7. PMID: 20102854, https://pubmed.ncbi.nlm.nih.gov/20102854/.

90. Foster GD, Linder B, Baranowski T, Cooper DM, Goldberg L, Harrell JS, et al. 2010. A school-based intervention for diabetes risk reduction. N Engl J Med. 363(5):443-53. PMID: 20581420, https://doi.org/10.1056/NEJMoa1001933.

91. Coleman KJ, Shordon M, Caparosa SL, Pomichowski ME, Dzewaltowski DA. 2012. The healthy options for nutrition environments in schools (Healthy ONES) group randomized trial: using implementation models to change nutrition policy and environments in low income schools. Int J Behav Nutr Phys Act. 27;9:80. PMID: 22734945, https://doi.org/10.1186/1479-5868-9-80.

92. Williamson DA, Champagne CM, Harsha DW, Han H, Martin CK, Newton RL Jr, et al. 2012. Effect of an environmental school-based obesity prevention program on changes in body fat and body weight: a randomized trial. Obesity (Silver Spring) 20(8):1653-61. PMID: 22402733, https://doi.org/10.1038/oby.201 2.60.

93. Bogart LM, Elliott MN, Cowgill BO, Klein DJ, Hawes-Dawson J, Uyeda K, et al. 2016. Two-Year BMI Outcomes From a School-Based Intervention for Nutrition and Exercise: A Randomized Trial. Pediatrics 137(5):e20152493. PMID: 27244788, https://doi.org/10.1542/peds.2015-2493.

94. Davis JN, Pérez A, Asigbee FM, Landry MJ, Vandyousefi S, Ghaddar R, et al. 2021. School-based gardening, cooking and nutrition intervention increased vegetable intake but

did not reduce BMI: Texas sprouts - a cluster randomized controlled trial. Int J Behav Nutr Phys 18(18):1-14. https://doi.org/10.1186/s1296 6-021-01087-x.

95. Patel AI, Schmidt LA, McCulloch CE, Blacker LS, Cabana MD, Brindis CD, et al. 2023. Effectiveness of a School Drinking Water Promotion and Access Program for Overweight Prevention. Pediatrics 152(3):e2022060021. PMID: 375 45466, https://doi.org/10.1542/peds.2022-060021.

96. Higgins JPT, Thomas J, Chandler J, Cumpston M, Li T, Page MJ, et al. 2023. Cochrane Handbook for Systematic Reviews of Interventions version 6.4 (update August 2023). Cochrane. https://training.cochrane.org/handbook [accessed 15 November 2023].
